# Genomic basis of environmental adaptation in the widespread poly-extremophilic *Exiguobacterium* group

**DOI:** 10.1093/ismejo/wrad020

**Published:** 2024-01-10

**Authors:** Liang Shen, Yongqin Liu, Liangzhong Chen, Tingting Lei, Ping Ren, Mukan Ji, Weizhi Song, Hao Lin, Wei Su, Sheng Wang, Marianne Rooman, Fabrizio Pucci

**Affiliations:** College of Life Sciences, Anhui Normal University, Wuhu 241000, China; Anhui Provincial Key Laboratory of Molecular Enzymology and Mechanism of Major Diseases, and Anhui Provincial Engineering Research Centre for Molecular Detection and Diagnostics, Anhui Normal University, Wuhu 241000, China; Center for the Pan-Third Pole Environment, Lanzhou University, Lanzhou 730000, China; College of Life Sciences, Anhui Normal University, Wuhu 241000, China; College of Life Sciences, Anhui Normal University, Wuhu 241000, China; College of Life Sciences, Anhui Normal University, Wuhu 241000, China; Center for the Pan-Third Pole Environment, Lanzhou University, Lanzhou 730000, China; Centre for Marine Bio-Innovation, University of New South Wales, Sydney, NSW 2052, Australia; School of Life Science and Technology, University of Electronic Science and Technology of China, Chengdu 611731, China; School of Life Science and Technology, University of Electronic Science and Technology of China, Chengdu 611731, China; Shanghai Zelixir Biotech Company Ltd., Shanghai 200030, China; Computational Biology and Bioinformatics, Université Libre de Bruxelles, Brussels 1050, Belgium; Interuniversity Institute of Bioinformatics in Brussels, Brussels 1050, Belgium; Computational Biology and Bioinformatics, Université Libre de Bruxelles, Brussels 1050, Belgium; Interuniversity Institute of Bioinformatics in Brussels, Brussels 1050, Belgium

**Keywords:** genomics, ecological units, poly-extremophile, Exiguobacterium, protein structure

## Abstract

Delineating cohesive ecological units and determining the genetic basis for their environmental adaptation are among the most important objectives in microbiology. In the last decade, many studies have been devoted to characterizing the genetic diversity in microbial populations to address these issues. However, the impact of extreme environmental conditions, such as temperature and salinity, on microbial ecology and evolution remains unclear so far. In order to better understand the mechanisms of adaptation, we studied the (pan)genome of *Exiguobacterium*, a poly-extremophile bacterium able to grow in a wide range of environments, from permafrost to hot springs. To have the genome for all known *Exiguobacterium* type strains, we first sequenced those that were not yet available. Using a reverse-ecology approach, we showed how the integration of phylogenomic information, genomic features, gene and pathway enrichment data, regulatory element analyses, protein amino acid composition, and protein structure analyses of the entire *Exiguobacterium* pangenome allows to sharply delineate ecological units consisting of mesophilic, psychrophilic, halophilic-mesophilic, and halophilic-thermophilic ecotypes. This in-depth study clarified the genetic basis of the defined ecotypes and identified some key mechanisms driving the environmental adaptation to extreme environments. Our study points the way to organizing the vast microbial diversity into meaningful ecologically units, which, in turn, provides insight into how microbial communities adapt and respond to different environmental conditions in a changing world.

## Introduction

High-throughput sequencing combined with metagenome binning and cultivation-dependent methods continues to expand the tree of life and Earth’s microbiomes [1, 2]. Complete domain-to-species taxonomic assignment has revealed the huge diversity of genomes and of metagenome-assembled genomes (MAGs) [3]. Plants and animals on Earth are easily classified into ecological units, but mapping the vast genomic diversity of microorganisms into ecologically meaningful units is still challenging [4-6]. Furthermore, lack of ecotype information hampers discovery of the genomic traits associated with environmental adaptation and prediction of how microbes respond to environmental change [7-9].

Multiple bacteria and archaea, such as *Vibrio* spp. [10], *Sulfolobus* spp. [11], *Prochlorococcus* spp. [12], and *Ruminococcus* spp. [6], have been used as models for microbial ecology studies. These studies have delineated particle-associated and free-living ecotypes in marine *Vibrio cyclitrophicus* [10], blue and red groups in hot spring *Sulfolobus islandicus* [11], and health- and disease-associated ecotypes in *Ruminococcus* [6].

However, the habitats of these organisms do not span the whole variety of biological ecosystems that characterize *Exiguobacterium*. Indeed, *Exiguobacterium* (Firmicutes, Bacilli, Exiguobacterales, and Exiguobacteraceae) is a poly-extremophilic genus whose members have frequently been detected and isolated from very diverse habitats, such as soils, desert, saline sediments, marine water, permafrost (3 million years old), glaciers, industrial products, and hydrothermal vents, with growth temperatures ranging from −12 to 55°C, pH from 5 to 12, and salinity from 0% to 19% (NaCl, m/v) [13-15]. The great genetic and habitat diversity of *Exiguobacterium* makes it an ideal model for studying the genomic basis of adaptation to different selective environments [13, 16, 17].

The ability to use and manipulate microbial processes has many potential applications, especially in extremophiles, whose functional abilities and evolutionary trajectories are not seen elsewhere in the microbial world [18]. *Exiguobacteria* are also interesting because of their very wide range of biotechnological applications. For example, enzymes from psychrophilic *Exiguobacteria* typically achieve high activity at low temperature at the expense of heat stability, thus providing a variety of natural resources of enzymes that function effectively in the cold such as chitinase from *Exiguobacterium antarcticum* DW2^T^ that is active at 0°C [19]. This intrinsic characteristic makes such enzymes valuable for many applications, e.g. in food, textile, clearing, environmental, and temperature-sensitive vaccine industries [20-22]. Moreover, the ability of some *Exiguobacterium* species to live in extreme conditions in terms of environmental pollutants is also of industrial interest, as their enzymes can be used in bioremediation and degradation of toxic substances [17, 23]. More precise and detailed sequence and structure information for the *Exiguobacterium* pangenome would not only help in understanding stress responses under extreme environmental conditions, and their link to protein architecture, but also improve the value of *Exiguobacterium* enzymes for biotechnology.

Microbial species use several complex strategies to adapt to the evolving environment, among which genetic adaptation and phenotypic plasticity [24, 25]. The first is a long-term adaptation mechanism through genetic modifications in the population and selection of phenotypes that are fittest in the new environment [26]. The second strategy is based on the ability of a given genotype to express different phenotypes and basically consists in expressing those that are best adapted to the environment. It is a fast-adaptation mechanism that is reversible and usually does not involve genetic change [26, 27]. The understanding of the interplay between phenotypic plasticity and genetic selection is a longstanding challenge in evolutionary biology [26, 28].

Our study is primarily focused on genetic adaptation, which is particularly fast in bacteria owing to their short generation times, even though it remains slower than phenotypic plastic modifications. We hypothesize that microbial diversity across different habitats, regardless of geographical location, can be delineated into ecologically meaningful units that are well separated in terms of genomic features. To test our hypothesis, we used here a reverse-ecology approach that leverages several levels of genomic information, with the aim of uncovering new perspectives on adaptation and of identifying genetic markers associated with different extreme environments. For this purpose, we sequenced eight type strains and utilized the 100 *Exiguobacterium* genomes, including 11 type strains, which were already available to assess the traits that explain adaptation of the genus to different extreme environments, and, more specifically, to hypersaline sediments, permafrost, glaciers, and hydrothermal vents.

We would like to emphasize that our novel reverse-ecology approach integrates for the first time different, complementary, genomic information layers. Indeed, following a comprehensive phylogenomic analysis of the *Exiguobacterium* pangenome, we performed in-depth analysis of regulatory elements that play a key and unexplored role in *Exiguobacterium* environmental adaptation. We then focused on protein sequences and their 3D structures. Although the vast majority of pangenome analyses are limited to protein sequences, protein structure is another essential level of information for understanding the biological functions of proteins and how they adapt to different environments. Here we took advantage of the recent development of AlphaFold2 [29], an artificial intelligence-based tool that predicts the structure of proteins from their sequence, with good accuracy and high throughput [29, 30]. Using this tool, we modeled the structure of all 230 000 protein sequences in the *Exiguobacterium* pangenome and integrated this information into the delineation of ecological units, with the aim of explaining adaptation to cold, hot, and hypersaline environments from a structural perspective.

## Materials and methods

### Collection of type strains and genome sequencing

At the time of this study (September 2020), 19 type strains of *Exiguobacterium* had been reported with valid published names (https://www.bacterio.net/). Of these 19 type strains, 11 had publicly accessible genomes and eight had no available genomes. We purchased these eight type strains from the China General Microbiological Culture Collection Center (*Exiguobacterium aestuarii* CGMCC 1.6140^T^ and *Exiguobacterium alkaliphilum* CGMCC 1.6140^T^), the German Collection of Microorganisms and Cell Cultures (*Exiguobacterium mexicanum* DSM 16483^T^, *Exiguobacterium artemiae* DSM 16483^T^, and *Exiguobacterium profundum* DSM 17289^T^), and the Japanese Collection of Microorganisms (*Exiguobacterium himgiriensis* JCM 14260^T^, *Exiguobacterium soli* JCM 14376^T^, and *Exiguobacterium aquaticum* JCM 17977^T^). All strains were recovered according to the instructions of their respective collection center.

Genomic DNA was extracted from isolates using a TIANamp Bacteria DNA kit (Tiangen, Beijing) following the manufacturer’s instructions. Using genomic DNA of the eight type strains, paired-end libraries with an insert size of 500 bp were constructed and sequenced using a HiSeq 2000 System (Illumina). Before de novo sequence assembly, low-quality reads were filtered out using Fastp with default options [31]. Filtered sequencing reads were assembled using SPAdes v.3.13.1 with default options [32]. The assembled genome sequences were deposited in DDBJ/ENA/GenBank under BioProject PRJNA862670.

### Preparation of *Exiguobacterium* genomes for analysis

In September 2020, we retrieved all *Exiguobacterium* genome sequences from GenBank, which provided 100 genomes, including 11 genomes of type strains and 21 MAGs. By adding the eight newly sequenced type strain genomes, we obtained a total of 108 genomes.

The genome is an integral part of an organism’s biological information; therefore, the more complete the genome, the more informative it is. However, too many gaps and contamination by fragments from other genomes (e.g. loss of information about gene order and missing genes that affect operon and functional makeup) limit the value of these genomes [33]. To circumvent this limitation, the 108 raw genomes were subjected to quality control and deduplication as follows: (i) contigs and N50 calculation using QUAST v.4.6.1 [34], (ii) completeness calculation of each genome using CheckM v.1.0.7 with default options [35], (iii) removal of genomes of >300 contigs, with N50 < 20 kb, completeness <95% and contamination >5%, and (iv) deduplication of genomes to remove those with amino acid identity (AAI) ≥ 99.5%. AAI values were calculated using CompareM with default options [35]. A total of 78 genomes met the quality requirements, which included all eight genomes of the newly sequenced type strains (Table S1).

### Phylogenomic and genomic analyses

The small subunit ribosomal RNA (16S rRNA) gene is widely used in bacterial phylogenetic classification and identification. It is multiply copied in about 30% of *Exiguobacterium* isolates (Table S1) and, because of its inter-diversity within the same strain, alleles of 16S rRNA genes are not usually monophyletic, resulting in mobile positioning of a strain in the phylogenetic tree based on 16S rRNA gene sequence (e.g. *Exiguobacterium acetylicum* DSM 20416^T^; Fig. S1). For phylogenomic clustering, a maximum likelihood tree was constructed using PhyloPhlAn3 with default options [36] ensuring that each of the isolates had a relatively fixed position in the tree. *Bacillus idriensis* DSM 19097 (GCA 009674765.1) and *Bacillus indicus* LMG 22858 (GCA 000708755.2) were chosen as the outgroup, as *Bacillus* is closely related to *Exiguobacterium*. Indeed, outgroup species that are closely related to ingroup species are more suitable for phylogenetic reconstruction [37-39]. The 78 genomes were first annotated using PROKKA v.1.14.5 with default options [40].

Carbohydrate-active enzyme and substrate were predicted using dbCAN2 and dbCAN3 with default options [41]. Genome-wide amino acid composition was calculated using CompareM with default options [35]. As the functions annotated by PROKKA are very detailed and difficult to interpret, genome-scale reconstruction of metabolic pathways and biogeochemistry profiles was further performed by gapseq v.1.2 [42] and METABOLIC v.4.0 with default options [43]. Horizontal gene transfer events were identified using MetaChip with 337 reference genomes (the 337 genomes were randomly selected from each species in the GTDB database release 95) from the order Bacilli [44, 45].

Promoters were predicted using Promotech v.1.0 with arguments -pg -m RF-HOT to parse the genomes, and -g -t 0.6 to predict promoter sequences; all other options were set to default [46]. Insertion sequences were predicted using ISEScan v.1.7.2.3 in default mode [47]. Small RNAs (sRNAs) were predicted using the standalone version of PredGsRNA in default mode [48]. Operons were predicted using the online version of Operon-mapper [49]. Results from gapseq, METABOLIC, Promotech, ISEScan, PredGsRNA, and Operon-mapper were parsed by custom R or Python scripts, which are available in our repository at github.com/environmental-genomes/Exiguobacterium.

### Growth temperature and salinity response

For growth temperature test, three replicates were grown in 100 mL of tryptic soy broth (TSB; Hope Bio-Technology) broth in 150 mL flasks at 50 and −1°C. For cultivation at −1°C, flasks were placed in ice produced by an ice maker (TKKY, FM40) with flasks placed in a ~4°C refrigerator and ice replaced every 12 h. Growth at 50°C was performed using a constant-temperature incubator. We adjusted the salinity of TSB to 3%, 5%, and 10% to perform the growth salinity test at 25°C. The optical density was measured at 600 nm (OD_600_) using a Microplate Reader (MD, SpectraMax M5) by transferring 200 μL of the culture into microwells.

### Protein sequence analysis

To study the properties of amino acid conservation within families of homologous proteins belonging to the *Exiguobacterium* pangenome, we considered all protein sequences obtained from the genomes of all *Exiguobacterium* strains using the annotation-based PROKKA program [40]. Then we used MMseq [50] with cut-off values of 0.50 on sequence identities and of 0.80 on sequence coverage to cluster all these proteins. The protein families thus obtained were better defined than those derived from PROKKA [40] (data not shown). For the conservation analyses, we considered only the families with at least 39 entries, which is half the number of genomes in our analysis. We then aligned the families thus obtained using ClustalW [51] and used SQUID (eddylab.org/software.html) for computation of the averaged sequence identity within families.

### Protein structure modeling

We modeled the structure of all the proteins of the entire *Exiguobacterium* pangenome. This represents a total of 239 724 proteins, from which we removed the few proteins whose sequence was partially undetermined. To limit the computational cost of structure modeling, we first clustered the entire protein set based on their sequence identity; we then used *ab initio* techniques for modeling the cluster representatives and homology modeling techniques for the other members of the clusters.

The protein clustering was performed using the MMseq software [50] with a cut-off value of 0.90 for both sequence identity and coverage. This led to a total of 34 524 protein clusters with at least one sequence picked up randomly from each cluster, and which have been modeled using the attention-based deep-learning method AlphaFold2 [29]. The total number of proteins modeled via AlphaFold2 was 65 704, consisting of about two sequences per cluster on average. The remaining 173 358 entries were modeled with the homology-based tool MODELLER [52] using as a structural template one of the structures predicted with AlphaFold2 in the cluster to which they belong.

### Protein structure analysis

We used in-house developed tools [53] to assign the per-residue secondary structure and solvent accessibility, defined as the ratio between the solvent-accessible surface area in the given 3D structure and in the extended Gly-X-Gly tripeptide conformation. Core residues are defined as having a solvent accessibility of at most 20% and surface residues, bigger than 20%. For identification of residue–residue interactions, namely, aromatic–aromatic, aromatic–sulfur, cation–π, ionic, disulfide, and hydrophobic interactions, we used ProtInter [54] with default options for the choice of the distance thresholds. Protein strengths and weaknesses, defined as regions that are particularly optimized or not optimized at all, respectively, for structural stability, were identified using the SWOTein software [55], which is based on the formalism of statistical potentials.

## Results

### Genome sequencing to cover all *Exiguobacterium* type strains

Knowledge of the pangenome of a given microorganism and its evolution is an important resource for ecological research, but is sometimes hampered by lack of availability of the genomes of all type strains (type strains are generally well matched to underlying contextual physiological data). In the case of *Exiguobacterium*, 19 type strains were well described but the genomes for eight of them were unavailable at the time of writing: *E. aestuarii*^T^, *E. alkaliphilum*^T^, *E. aquaticum*^T^, *E. artemiae*^T^, *E. himgiriensis*^T^, *E. mexicanum*^T^, *E. profundum*^T^, and *E. soli*^T^ (see Materials and Methods for details). We purchased these eight strains and sequenced their genomes, so the genomes of all 19 *Exiguobacterium* type strains are now available (Table S1). According to the rules of the *International Journal of Systematic and Evolutionary Microbiology* [56], genome sequence is mandatory for the taxonomy of prokaryotes.

### Phylogenomics analysis of *Exiguobacterium*

The phylogeny relationships of a total of 78 nonredundant high-quality *Exiguobacterium* genomes, of which eight were MAGs, 19 were assembled from type strains, and 51 from non-type strains (Table S1) whose collection and preparation are described in Materials and Methods, were analyzed by constructing a maximum likelihood phylogenomic tree based on the 400 most universal markers [36].

**Figure 1:**
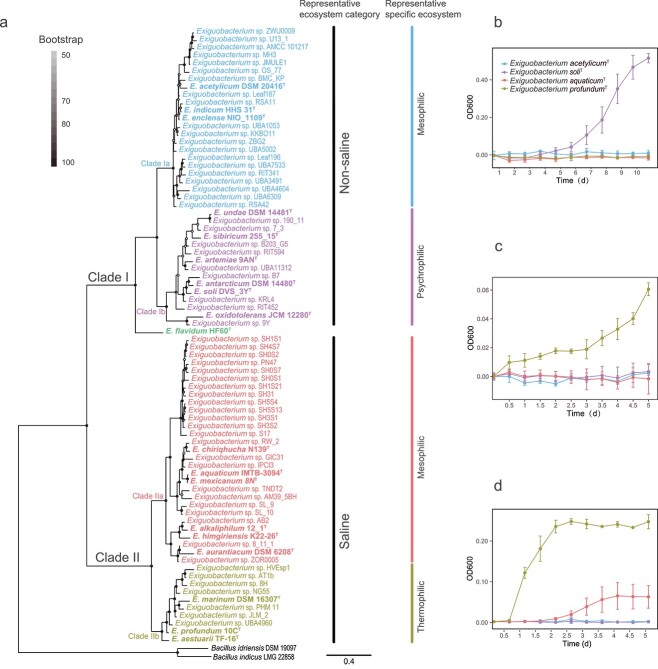
*Exiguobacterium* phylogeny and growth profiling. (A) Maximum likelihood *Exiguobacterium* phylogenomic tree and representative ecosystem classification. The tree has two major clades, the nonsaline Clade I and the saline Clade II; and four subclades, the nonsaline mesophilic Clade Ia, the nonsaline psychrophilic Clade Ib, the saline mesophilic Clade IIa, and the saline thermophilic Clade IIb. Bar, 0.4 substitution per amino acid position. (B–D) Growth curves for representative isolates of Clade Ia (blue symbols and line, *E. acetylicum*^T^), Clade Ib (purple symbols and line, *E. soli*^T^), Clade IIa (orange symbols and line, *E. aquaticum*^T^), and Clade IIb (olive green symbols and line, *E. profundum*^T^) at (B) −1°C, (C) 50°C, and (D) 10% NaCl (m/v) at 25°C. All growths were measured with tryptic TSB as basal medium.

The *Exiguobacterium* were separated into two main clades, which branched from the root of the tree (Fig. 1A). The upper clade, designated as Clade I, is composed of genomes from mainly nonsaline environments (soil, plants, permafrost, and glaciers; Fig. 1A, Tables 1 and S1). The lower clade, designated as Clade II, is composed of genomes from saline environments (saline lake, tidal flat, and hydrothermal vents; Fig. 1A, Tables 1 and S1). Note that type strain *Exiguobacterium flavidum* HF60^T^ isolated from freshwater of Red Maple Lake [15] was not grouped in either of these two clades, and is located at the interface between Clades I and II.

**Table 1:** Average of genomic features in the different *Exiguobacterium* subclades: *n*, number of isolates in each subclade; Ts, type strains; note that type strain *E. flavidum* HF60^T^ was not in any of the four clades.

		Size (Mb)	CDS	Coding Density (genes per kb)	CRISPR	GC content (%)
Clade Ia	*n* = 23 (6 MAGs, 3 Ts)	3.11 (± 0.15)	3204 (± 145)	0.89 (± 0.01)	0.26 (± 0.54)	47.0 (± 0.3)
Clade Ib	*n* = 15 (1 MAGs, 6 Ts)	3.07 (± 0.13)	3117 (± 127)	0.88 (± 0.01)	0.47 (± 0.83)	47.3 (± 0.4)
Clade IIa	*n* = 29 (6 Ts)	2.92 (± 0.09)	2997 (± 106)	0.89 (± 0.01)	0.97(± 1.27)	52.1 (± 0.8)
Clade IIb	*n* = 10 (1 MAGs, 3 Ts)	2.90 (± 0.13)	2964 (± 140)	0.90 (± 0.01)	0.80 (± 0.92)	48.1 (± 0.4)

The phylogenomic information suggests that the nonsaline Clade I can be further separated into two subclades, Clades Ia and Ib (Fig. 1A). One of these subclades (Ib) harbors isolates from mainly cold stressful habitats (i.e. permafrost and glaciers) that can grow at temperatures below 0°C, whereas Subclade Ia harbors mesophilic isolates mainly from mesophilic benign environments. The saline Clade II is also divided in two subclades, IIa and IIb, with the latter harboring isolates from mainly hot environments (hot springs and hydrothermal vents) that can grow at high temperature (Fig. 1A), with Clade IIa harboring mesophilic isolates.

We analyzed the mean annual temperature (MAT) of the isolation source for the different subclades and found a clear separation between them. Indeed, the average MAT is equal to −1.6°C for cold adapted Clade Ib, to 8.9and 12.1°C for mesostable Clades Ia and IIa, respectively, and to 45.5°C for hot-adapted Clade IIb (see Table S1 for more details). Finally, an equally clear separation has been found between the mean salinity of the isolation source of Clade I (<1% NaCl, m/v) and Clade II (>5% NaCl, m/v) (Table S1).

To experimentally verify the identification of ecological units, we performed growth curve tests for representative isolates of Clade Ia (*E. acetylicum*^T^), Clade Ib (*E. soli*^T^), Clade IIa (*E. aquaticum*^T^), and Clade IIb (*E. profundum*^T^) under different growth conditions (see Fig. 1B–D). Specifically, the lower temperature limit (−1°C) is the definitive difference between Clade Ib isolates, which can grow at this temperature, and Clade Ia isolates, which cannot. The upper temperature limit (45°C) is the definitive difference between Clades IIa and IIb isolates, with the latter being able to grow under these conditions, whereas the former cannot.

Growth curves under different salinity conditions (3%, 5%, and 10% NaCl at 25°C, m/v) clearly differ between Clades I and II, with the latter growing much better than the former in saline conditions. Moreover, Subclades Ia and Ib have similar growth curves at any of the salinities tested. In contrast, Clades IIa and IIb show an almost identical growth at 3% salinity; Subclade IIa becomes less able to grow than Subclade IIb at 5% although it still grows better than nonsaline Clade I; only Subclade IIb is able to grow at more extreme environment of 10% salinity (see Figs 1 and S2).

### Genomic features of *Exiguobacterium*

The general genomic characteristics, and more precisely the genome size, GC content, codon usage, and clustered regularly interspaced short palindromic repeats (CRISPR) loci, further support classification of *Exiguobacterium* into two main clades and four subclades, as shown below.

**Figure 2:**
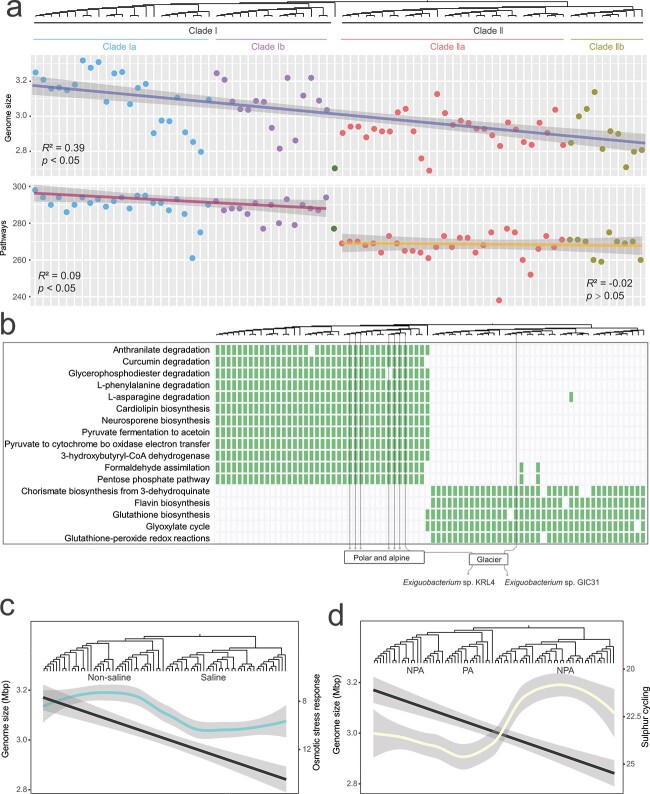
Genome content and specific metabolic pathways of *Exiguobacterium* clades and subclades. (A) The change in the number of metabolic pathways between Clades I and II is more abrupt than the change in genome size as shown by the linear regression of genome size and the number of pathways with respect to the position in the phylogenomic tree. (B) Specific metabolic pathways in Clades I and II and glacier isolates located in Clades Ib and IIa have distinct specific pathways, which are consistent with their phylogenomic status rather than isolation sources. For example, strain *Exiguobacterium* sp. KRL4 and *Exiguobacterium* sp. GIC31, both isolated from glaciers, have different specific pathways. Green blocks indicating the presence of a pathway. Loess fitting curves of the number of (C) osmotic stress response-related genes (cyan line, referring to right *y*-axis) and (D) sulfur cycling-related genes (yellow line, referring to right *y*-axis), with the node of the host isolates ordered according to their position in the phylogenic tree. The black lines (referring to the left *y*-axes) in panels (C) and (D) correspond to the genome size regression line shown in panel (A).

The size of the *Exiguobacterium* genomes ranges from 2.69 to 3.32 Mbp, with an average of 3.00 Mbp. The genome size increases from the root to the deep branches of the *Exiguobacterium* tree, resulting in larger genome sizes in the terrestrial Clade I (average of 3.10 Mbp) than in the saline Clade II (2.92 Mbp) (Table 1, Fig. 2a). These results are in agreement with the genome size distribution analysis performed by [57], in which microbial genomes of terrestrial ecosystems have been shown to be larger than those of aquatic environments. There is no significant difference in genome size between Subclades Ia and Ib, and between Subclades IIa and IIb (*P* > .05, Wilcoxon).

**Figure 3:**
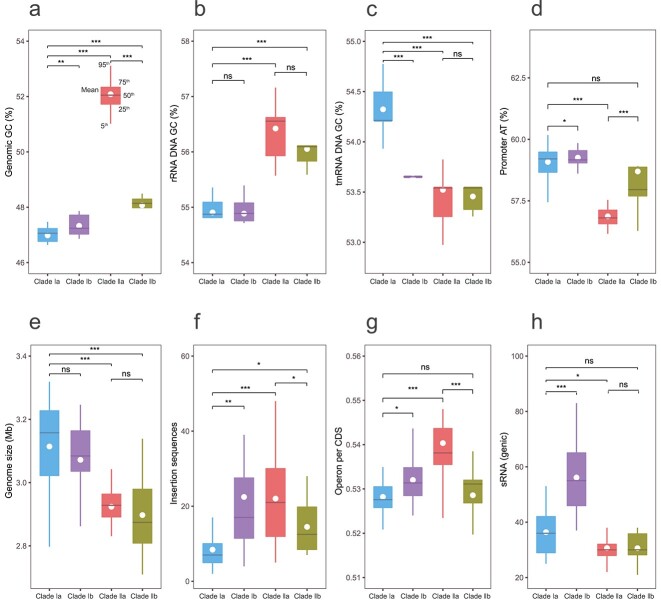
Characteristics of regulatory elements of the four *Exiguobacterium* subclades in comparison with general genomic features. Box plots of (A) GC content in the whole genome; (B) GC content in the rRNA gene; (C) GC content in the tmRNA gene; (D) AT content in promoters; (E) genome size; (F) insertion sequence; (G) operon per CDS; (H) sRNA content. ns, not significant, *P* > .05; ^*^P < .05; ^*^^*^P < .01; ^*^^*^^*^P < .001.

The genome-wide GC content of *Exiguobacterium* isolates and MAGs ranges from 45.9% to 55.0%, with an average of 49.2%. Clade I has a significantly lower GC content than Clade II, in agreement with the higher GC content of halophilic organisms [58] (Table 1, Fig. 3A, *P* < .05, Wilcoxon). The GC content of Clades Ia and Ib is almost identical, whereas Clade IIa has a much higher GC content than Clade IIb. This result is consistent with the lack of universal relationship between genome GC content and growth temperature profile of prokaryotes [59], but inconsistent with recent data pointing to a positive correlation between these quantities [60]. Focusing on the GC content of the codons, we observe that the distribution of GC at the first codon position (GC1) follows a similar behavior to the genome-wide GC content, but with stronger relative differences between the clades. GC2 and GC3 are substantially different, with smaller separations between the clades (Fig. S3A–D).

Changes in GC content are often taken a sign of horizontal gene transfer [61, 62] events. We detected a total of 74, 55, 80, and 65 such events in Subclades Ia, Ib, IIa, and IIb using Bacilli genomes as a reference (Fig. S4). This suggests that the high GC content in Clade IIa could be partly related to frequent horizontal gene transfer events. In contrast, the low GC content in Subclades Ia and Ib does not agree with their number of horizontal gene transfer events, with Ia having substantially more events than Ib [63].

We then analyzed codon usage information of isolates by performing a dimensional reduction with a nonmetric multidimensional scaling (NMDS) approach. The results (Fig. S5A) show that subclades separate well in the resulting low dimensional space, thus indicating that, interestingly, the modulation of codon usage is another factor used by microorganisms for environment adaptation [64].

CRISPR loci are a type of memory of encountered foreign invaders and allow a faster adapted immune response in the case of reinvasion [65]. We found important CRISPR loci differences between the different clades, with Subclade Ib having almost double the number of CRISPR loci compared with Clade Ia, and with Clades IIa and IIb having four and three times the number of loci compared with Clade Ia, respectively (Table 1). This suggests that saline *Exiguobacteria* have probably experienced more phage invasion than nonsaline ones [66]. The number of CRISPR loci is also increased in psychrophilic clades compared with mesophilic ones. However, this trend differs from what has been observed in a previous study in which the authors suggested a linear correlation between temperature (and oxygen concentration) and CRISPR incidence [67].

In summary, the phylogenomic and genomic characteristics, as well as the isolation information, support the classification of the *Exiguobacterium* into two main clades, terrestrial Clade I and saline aquatic Clade II, which can be further divided into four subclades: mesophilic Clade Ia and psychrophilic Clade Ib, and mesophilic saline Clade IIa and thermophilic saline Clade IIb. The four subclades have an equal average branch length < 0.5 to the leaf taxa (Fig. S5B), suggesting that isolates in each clade have the same evolutionary distance [1].

The type strain *E. flavidum* HF60^T^ has general genome characteristics that differ from those of both Clades I and II, indicating that *E. flavidum*^T^ may represent a third main clade (freshwater Clade III, Fig. S5B) of *Exiguobacterium*. However, it is hard to describe the overall genome features of this potential clade based on a single genome. It has been suggested that at least three nonredundant genomes are needed to describe the genomic features of a lineage [68].

### Habitat-specific function and adaptation

Variation of functional capacity and gene content between clades with a common ancestor can inform habitat-specific adaptation [6, 69, 70]. We thus analyzed the distribution of about 4500 functional genes identified by PROKKA [40] in the 78 isolate genomes and MAGs. To obtain insightful information from these data, we performed a NMDS analysis and plotted the results (Fig. S5C) in which we observed a clear separation of the different subclades. This indicates that habitat-specific functional genes are of fundamental importance to make organisms suited for living in their habitats.

We observed a notable higher number of metabolic pathways in Clade I than in Clade II, which cannot solely be attributed to the larger genome sizes (Fig. 2A). Moreover, we identified 12 and 5 habitat-specific functions in Clades I and II *Exiguobacterium*, respectively; these functions are likely to reflect the adaptation of the two main ecological types (Fig. 2B).

Four of the 12 Clade I-specific functions were related to carbohydrate and amino acid degradation, such as degradation of curcumin (a polyphenol produced by terrestrial plants of the *Zingiberaceae* family) [71]. The Clade II-specific functions were mainly related to increasing the efficiency of carbohydrate utilization to overcome the inhibitory effect of high salinity, such as the glyoxylate cycle [72]. The presence of specific carbohydrate-active enzyme (CAZyme) genes can also differentiate between the two major clades with, e.g. the polysaccharide lyase 9 (PL9_2, involved in utilization of plant carbohydrates) and the glycosyltransferase family 26identified exclusively in Clade I [73], whereas the carbohydrate-binding module family 41 and the carbohydrate esterase families 13 and 14 are found only in Clade II (see Fig. S6A). The functional differentiation of *Exiguobacterium* Clades I and II reflects the adaptation to their respective environments and is a further indication of the close relationship between bacterial phylogeny and habitat type [74]. We also analyzed in more detail the metabolic capacity of *Exiguobacterium* to use carbohydrate substrates. The result shown that they are able to use ~20 carbohydrate substrates such as beta-glucan, sucrose, starch, peptidoglycan, chitin, xylan, glycogen, beta-galactan, beta-fucosides, and polyphenol. NMDS analysis of the carbohydrate substrate utilization matrix showed that Clades I and II were clearly separated (Fig. S7A), and CAZymes linked with chitin utilization were more than twice as abundant in Clade II than in Clade I (6.74 vs. 3.00, Fig. S7B). In contrast, the two Subclades Ia and Ib overlap, as do Subclades IIa and IIb (Fig. S7A). This overlap in the overall metabolic capacity to utilize carbohydrate substrates between Clades Ia and Ib, and between Clades IIa and IIb, suggests that the C-source is not (auto)correlated with the differences found between saline soil/permafrost and glacier environments and between saline sediment/hydrothermal vent environments.

Gene enrichment and high level of redundancy can also reflect habitat-specific stresses [75, 76]. Using the overall trend of genome size as a control factor, genes related to the osmotic stress response and sulfur cycle were identified in the saline Clade II and psychrophilic Subclade Ib (see Fig. 2C and D). The enrichment of osmotic stress genes in Clade II agrees with the fact that most of the isolates of this clade came from saline lakes and thus tolerate higher salinity. The psychrophilic Subclade Ib was enriched in genes involved in the sulfur cycle such as *betB*, which is involved in the biosynthesis of the osmo- and cryo-protectant glycine betaine, and *ssuD*, which is involved in the acquisition of organic sulfur. This enrichment suggests that sulfur metabolism may be critical to bacteria that thrive in subzero temperatures and that these genes were under stronger positive selection in the psychrophilic isolates. A similar pattern of habitat-specific functions between Subclades Ia and Ib indicates that psychrophilic *Exiguobacterium* have comparable abilities in driving biogeochemical cycles in subzero temperatures, with, moreover, an enhanced sulfur cycle metabolism. Our results are consistent with the notion that polar- and alpine-specific microbes are strongly adapted to regulate sulfate cycling in cold environments [77, 78].

In summary, by consistently ordering the multiple *Exiguobacterium* genomes into sharply delineated ecological units and mapping functional traits to each unit, our work facilitates the link between specific microbial clades and particular ecological processes, which has proven to be difficult to achieve otherwise [79].

### Regulatory features of the extremophilic clades

Enrichment of specific genes and pathways, as well as optimization of tetranucleotide composition, is an important genomic underpinning for microorganisms to adapt to extreme environmental conditions [80, 81]. However, regulation of functional genes is also very important although poorly understood [82]. Here we analyzed this issue in more detail by exploring how differences in regulatory regions are related to the habitat of the ecological units defined above.

In ribosomal RNA (rRNA), we observed a globally higher GC content than at genome level (Fig. 3A and B, Table S2). However, the trends between clades are similar, with a higher GC content in the halophilic Clades IIa and IIb than in Clades Ia and Ib; note that the GC content is much bigger in Clade IIa than in IIb when considering the full genome, whereas the difference is relatively small in rRNA. Thus, we did not find the expected positive correlation between rRNA GC content and growth temperature [83].

Transfer-messenger RNA (tmRNA) recycles stalled ribosomes and contributes to the degradation of incomplete proteins, playing an important role in bacterial development and environmental stress response [84]. In this study, we identified a consistent decrease in the tmRNA GC content in all the extremophilic clades with respect to Clade Ia (Fig. 3C, Table S2). This behavior, which is opposite to that for genome GC content, suggests that tmRNA composition may be an overlooked but important indicator of adaptation shared by multiple types of extremophiles.

Promoters are AT-rich regions located upstream of genes, where the *σ* factor binds to initiate gene transcription [85]. An increase in AT content, because of substitution of C to T or G to A, results in improving the transcription efficiency of the promoter [86]. We identified the largest AT content in Clade I, with the psychrophilic Clade Ib only marginally more enriched in AT than Clade Ia. Halophilic Clade II has a significant lower promoter AT content, especially the mesophilic Clade IIa (Fig. 3D, Table S2). This observation suggests that enhancing transcriptional efficiency is important for low-temperature growth and that this feature can clearly distinguish psychrophilic Clade Ib from the other extremophilic Clades IIa and IIb.

Even though the extremophilic Subclades Ib, IIa, and IIb have smaller genomes than mesophilic Subclade Ia (Figs 2A and 3E, Table S2), they host more insertion sequences, as already documented [87] (Fig. 3F, Table S2). They also have higher proportions of operons per coding sequences (CDS; Fig. 3G, Table S2). Bacteria tend to arrange metabolically or functionally related genes into operons that are co-transcribed in the same polycistronic messenger RNA. Operons, such as the lactose operon and tryptophan (Trp) operon, enable bacteria to respond quickly and efficiently to changes in metabolite status and to other environmental parameters [88]. Higher densities of operons per CDS improve the fitness of extremophilic clades to adapt to their specific habitats. Note that, within Clade I, the psychrophilic Subclade Ib hosts more insertion sequences and has a higher proportion of operons than mesophilic Clade Ia, whereas, in saline Clade II, the thermophilic Subclade IIb has fewer insertion sequences and proportion of operons than its mesophilic counterpart IIa.

Bacterial sRNA regulators bind to target mRNAs or proteins to stabilize mRNAs and to modulate protein activity [89, 90]. Thus, sRNAs play an important role in the regulation of many cellular processes, such as the response to environmental changes through increased stress resistance or facilitated survival in various ecological contexts [91, 92]. One mechanism of sRNA to modulate physiological responses to environmental changes is to increase its copy number, which enables sRNAs to act redundantly or additively to increase the sensitivity and efficiency of a response [93, 94]. We observed a significant increase in the number of sRNA in the psychrophilic Clade Ib with respect to Clade Ia (Fig. 3H, Table S2), but not in the mesophilic saline Clade IIa nor the thermophilic saline Clade IIb. As the genome size of Clade Ib is smaller than that of Clade Ia, the increased sRNA copy number is likely to be a strong contributing factor of Clade Ib adaptation to cold glacier and permafrost ecological niches.

The RNA regulators are more frequent in the psychrophilic clade than in the other clades and this trend appears to be equally important for RNA as for protein regulators, such as cold shock proteins. Indeed, there is an enrichment in the number of cold shock genes in Clades Ib (Fig. S6B and C). Note that RNA regulators are less costly to the cell and can be faster to produce compared with protein regulators [89] and thus may be critical for bacteria growth in the frozen world, where energy and nutrients are limited.

In summary, a decrease in tmRNA GC content and an increase in the density of operons is shared by the psychrophilic Clade Ib, the saline Clade IIa and the hypothermal vent Clade IIb. This suggests that the efficiency in rescuing the ribosome, as well as the regulation of function-related gene clusters, is important for many kinds of extremophiles. We also found that the promoter AT content and the increasing number of sRNA are exclusive to the psychrophilic Clade Ib, suggesting that additional changes in genomic regulatory elements are needed to break through the limitations of low-temperature growth.

### Amino acid composition of proteins from *Exiguobacterium* clades

Besides gene regulation optimization, amino acid optimization is a basic strategy that allows proteins to function under different environmental conditions and is thus exploited by host organisms for environmental adaptation. For example, amino acid optimization allows proteins from halophilic microorganisms to remain soluble under saline conditions [95], or for proteins from cold-adapted organisms to remain active at temperatures close to 0°C [81, 96, 97].

We began by analyzing amino acid conservation within the families of homologous proteins from *Exiguobacterium*, as defined in Materials and Methods. In general, conservation within families is high: the per-family sequence identity is about 80% on average, with a standard deviation of about 8%. If we focus only on protein families belonging to either Clade I or Clade II, the average sequence identity increases up to about 90%, with a standard deviation of 9%. These high values indicate that just a few changes in protein sequence can drastically modify functional or biophysical properties and allow adaptation to very different environmental conditions. This astonishing adaptation ability of proteins is already known for proteins from other types of bacteria [98]. The mechanisms of adaptation are often family dependent [97, 99], which makes identification of the factors driving adaptation very complex.

**Figure 4:**
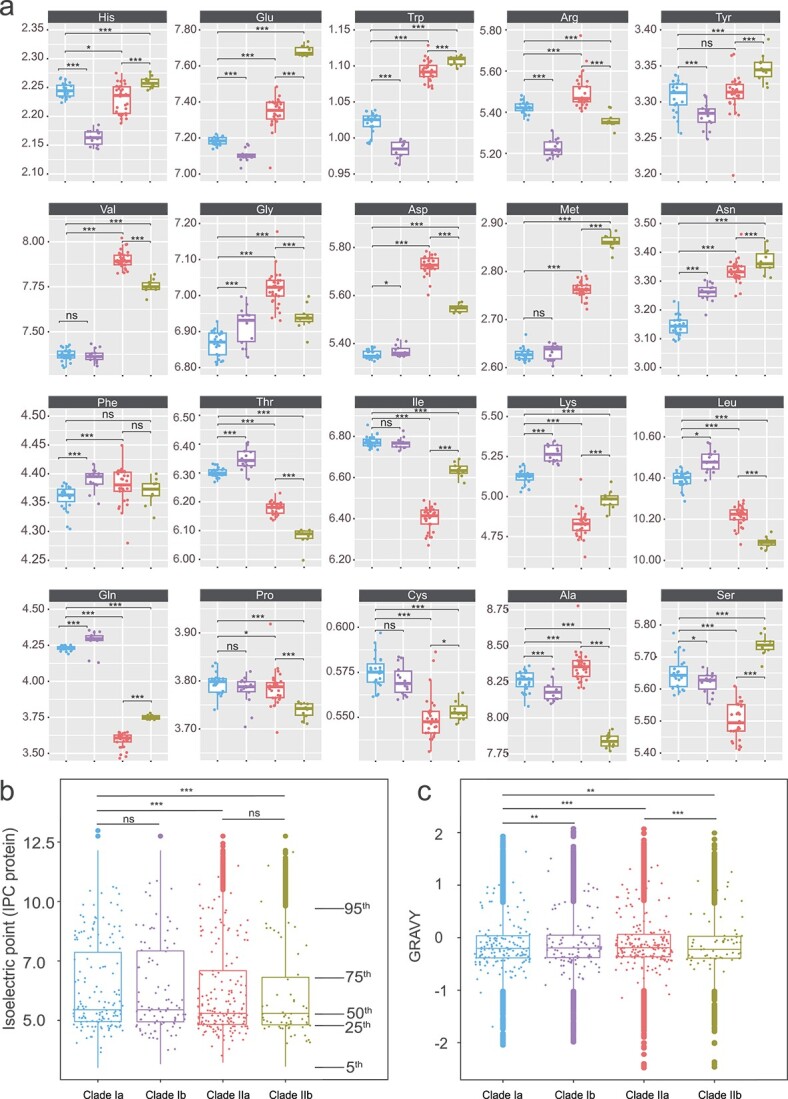
Changes in genome-wide amino acid composition reflecting salinity and temperature adaptation to different habitats of the four *Exiguobacterium* subclades. (A) Comparison of the composition of the 20 amino acids between the four subclades. (B) Plot of genome-wide isoelectric point showing that the saline-adapted Clade II has a lower isoelectric point than the nonsaline Clade I. (C) Plot of genome-wide GRAVY score (grand average of hydropathicity); Clades Ib, IIa, and IIb all have higher GRAVY scores than Clade Ia. ns, not significant, *P* > .05; ^*^P < .05; ^*^^*^P < .01; ^*^^*^^*^P < .001.

**Figure 5:**
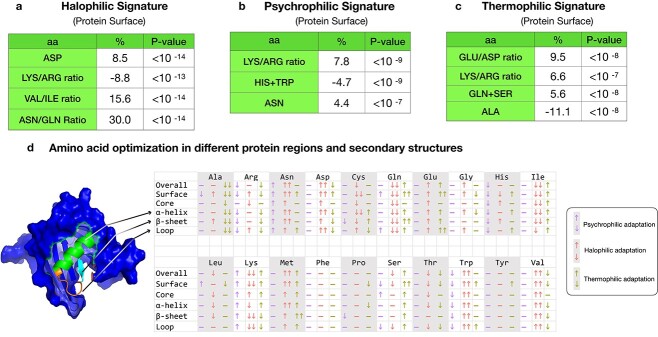
Summary of the main trends at protein level observed for the environmental adaptation of *Exiguobacterium*. (A–C) Percentage difference between the average amino acid frequency of a given clade relative to the reference clade. (A) Halophilic signature obtained by comparison of Clade IIa to Clade Ia. (B) Psychrophilic signature obtained by comparison of Clade Ib to Clade Ia. (C) Thermophilic signature obtained by comparison of Clade IIb to Clade IIa. *P*-values were obtained from the Kolmogorov–Smirnov test of the amino acid distribution in the different clades. (D) Amino acid percentage differences between clades when restricted to different protein regions and secondary structures. The three columns correspond to psychrophilic adaptation (Clades Ib vs. Ia), halophilic adaptation (Clades IIa vs. Ia), and thermophilic adaptation (Clades IIb vs. IIa). Arrows ↓↓ and ↓ correspond to a percentage decrease of more than −5% and between −1% and −5%, respectively. Arrows ↑↑ and ↑ correspond to a percentage increase bigger than 5% and between 1% and 5%; − indicates no significant change.

In what follows, we analyzed how the amino acid composition of *Exiguobacterium* proteins has been shaped to adapt to their different habitats. To facilitate the analysis of the factors that drive the environmental adaptation of *Exiguobacterium* isolates at protein level, the main trends observed are summarized in Figs 4 and 5 (further details are given in Figs S8–S9).

### Optimization for saline environments

To examine the effect of amino acid composition on saline adaptation, we compared the frequency of all 20 amino acids between mesophilic terrestrial Clade Ia and mesophilic saline aquatic Clade IIa. We found the frequencies of almost all amino acid types to be significantly different between the two clades (Fig. 4A, Table S2).

The negatively charged residues Asp and Glu show a significant increase of more than 5% in halophilic Clade IIa with respect to Clade Ia (Fig. 5A). This trend is known to be one of the key hallmarks of halophilic adaptation. Although the precise mechanisms are not yet totally clear, the fact that Asp and Glu side chains have very favorable free energies of hydration, much more favorable than positively charged residues [100], allows halophilic proteins to remain soluble even in high salinity conditions, and to avoid aggregation [101, 102]. The frequency increase of Asp, which has the most favorable free energy of hydration, is stronger than that of Glu and occurs preferentially at the surface of the proteins (see Fig. 5A and D). This is a further indication that enhanced solvation properties contribute to the adaptation to a high salinity environment.

A drop in lysine (Lys) frequency was also observed in Clade IIa compared with Clade Ia, especially in protein surface regions (Fig. 5A and D). This positively charged residue has a very long side chain, which has been shown to have less favorable hydration free energies and to be less favorable than other charged residues in an environment with low water content [103, 104].

Among the aliphatic residues valine (Val), isoleucine (Ile), and leucine (Leu), the latter two are significantly underrepresented in halophilic Clade IIa, and the former is overrepresented (Fig. 5A and D). This could be attributed to the smaller size of Val with respect to Ile and Leu, and is in agreement with earlier studies [105]. Among the polar residues, side chain size also seems to play a role: for the similar residues asparagine (Asn) and glutamine (Gln), the smallest one is much more frequent in halophilic Clade IIa and the largest one is strongly depleted (Fig. 5A and D). Another observation is the higher frequency of the hydrophobic residue methionine in halophilic Clade IIa, and of the aromatic residue Trp, with an enrichment of about 7%.

Note that all these trends are stronger on the surface than in the core of proteins (Figs 5D and S8–S9).

### Optimization for cold environments

To identify how amino acid composition facilitates protein adaptation to cold environments, we compared Clades Ib and Ia, which are composed of psychrophilic and mesophilic organisms, respectively.

The Lys/Arg ratio is a major signature that is observed in adaptation to cold. Indeed, Arg is known to confer a higher degree of stabilization to proteins than Lys, because it forms stronger ionic interactions. It is thus likely to be replaced by Lys in cold-adapted organisms [106, 107]. This trend is prevalent on the surface, with a Lys/Arg ratio for cold-adapted *Exiguobacterium* proteins that is 8% higher than that for mesophilic *Exiguobacterium* proteins (see Fig. 5B and D).

With the exception of Phe, the most hydrophobic of the aromatic residues, we found that the aromatic residues Tyr, Trp, and His are depleted in psychrophilic isolates, as has been pointed out by [107]. Loosing aromatic residues that often participate in interaction networks with positively charged and other aromatic residues can be seen as a mechanism to weaken the structure of cold-adapted proteins [108].

The final clear trend that we observe is the increase in the frequency of some polar amino acids, such as Asn, Thr, and to a lesser extend Gln, in psychrophilic Clade Ib. This effect arises from the surface (Fig. 5D) where an excess of polar residues provides a very hydrophilic surface, which results in stronger protein–solvent interactions, as well as in a reduction in the compactness of the protein [109].

### Optimization for hot environments

To identify how amino acid composition facilitates adaptation of proteins to hot environments, we compared Clade IIb with Clade IIa, which are composed of mesophilic and thermophilic organisms, respectively. In this way, we attempted to decouple the effect of the high salinity environment, in which all organisms from Clade II live, and the effect of high temperature, which characterizes only the habitat of Clade IIb whose isolates mainly come from hydrothermal vents. Note, however, that isolates from Clade IIb can live at higher salinity concentrations than those of Clade IIa. The decoupling is therefore only partial.

The first clear trend we observe from this comparison is a strong increase in Glu content with respect to Asp (Fig. 5C). This trend is a known signature of thermophilic adaptation [97, 110], and has been suggested to be because of the higher conformational entropy of Glu [111]. This trend occurs exclusively at the protein surface with an increase of Glu/Asp ratio of about 10% in mesophilic Clade IIa compared with thermophilic Clade IIb (Fig. 5C and D). Note that both Glu and Asp are much more frequent in Subclades IIa and IIb than in Clade Ia, as these subclades host isolates from saline aquatic environments.

The second trend is an increase of Lys with respect to Arg on the protein surface (Fig. 5C and D). This has been suggested to contribute to thermophilic adaptation, as Lys has a higher conformational entropy than Arg [112], and is thus likely to contribute more favorably to the protein free energy at high temperature.

We note some unexpected trends, with an increase in some polar amino acids (especially Gln and Ser) that is not in agreement with previous observations [110, 113]. These trends appear as a signature of Clade IIb when compared with Clade IIa, but deserve further investigation to better understand their role in the adaptation mechanisms. They could perhaps be explained by the joint effects of high-saline and high-temperature environments. Finally, we also observed an important depletion of Ala on the surface of thermophilic proteins (Fig. 5C).

### Isoelectric point and grand average of hydropathy index

We computed the isoelectric point of all proteins in the *Exiguobacterium* proteome and averaged these for each isolate (see Materials and Methods). A lower value of the isoelectric point was observed in Clade II compared with Clade I (Fig. 4B, *P* < .05, Wilcoxon); this is consistent with the fact that isolates from Clade II were mostly derived from saline environments. Between the subclades, no significant difference in isoelectric point was observed between Clades Ia and Ib. However, Clade IIb has a lower isoelectric point than Clade IIa, which is consistent with the fact that Clade IIb hosts isolates that are able to grow in much higher salinity conditions, with >15% NaCl (Fig. 4B).

We also computed the grand average of hydropathy (GRAVY) index, which is related to the hydrophobicity of the proteins, and averaged it for each isolate. We found that the GRAVY index was significantly lower in Clade II than in Clade I (Fig. 4C). This is explained by the fact that, as a general trend, halophilic organisms show less hydrophobic proteomes than mesophilic organisms [114]. This is probably aimed at counterbalancing the strengthening of the hydrophobic forces in the presence of high salt concentrations and thus avoiding aggregation phenomena in hypersaline environments.

### Structural and dynamical features of proteins from *Exiguobacterium* clades

We modeled the protein structures of the whole *Exiguobacterium* pangenome (containing about 230 000 proteins) using AlphaFold2 [29] and homology modeling [52] as described in Materials and Methods. This information allowed us to go deeper into the analysis of structural differences between the folded conformations of homologous proteins from halophilic, psychrophilic, mesophilic, and thermophilic *Exiguobacterium*. Note that, because of the high sequence similarity between clades, proteins from different isolates are highly conserved structurally, and the main differences are localized in surface regions, as noted in the previous section and seen in Fig. 5.

We began by analyzing the secondary structure composition, which is one of the features often used to explain environmental adaptation. For example, enzymes that remain active at low temperature tend to have longer loops than their mesostable counterparts, which allow increased protein flexibility and maintenance of catalytic activity [115-117]. On the other hand, deletion of exposed loop residues has been suggested to enhance protein thermostability [97, 99]. At the pangenome level, we found that the secondary structure composition is not statistically significantly different among the *Exiguobacterium* clades, and that the fraction of residues in alpha-helix, beta-sheet, and loop structures does not change.

We also analyzed the role of amino acid interactions in driving environmental adaptation of the *Exiguobacterium*. In more detail, we computed and compared the occurrence of salt bridges, hydrophobic interactions, and interactions involving aromatic residues (aromatic–aromatic interactions, cation–π interactions, and aromatic–sulfur interactions). The average number of these interactions divided by protein length is reported in Table 2 for the different *Exiguobacterium* subclades.

**Table 2:** Content of amino acid interactions divided by protein length and averaged over all proteins of the given subclade. The *P*-values obtained by the Kolmogorov–Smirnov test between the distribution of the interactions in the given subclades and the reference subclades are given in parentheses. Reference clades are Ia for clade Ib to analyze psychrophilic adaptation; Ia for IIa to analyze halophilic adaptation; Clade IIa for Clade IIb to analyze thermophilic adaptation.

	Salt bridges	Aromatic	Hydrophobic
Clade Ia	0.093 (—)	0.078 (—)	0.36 (—)
Clade Ib	0.091 (< 10^−20^)	0.077 (< 0.01)	0.362 (< 0.005)
Clade IIa	0.094 (< 10^−25^)	0.079 (< 10^−7^)	0.371 (<1 0^−25^)
Clade IIb	0.095 (< 10^−20^)	0.079 (< 0.001)	0.365 (< 10^−25^)

Salt bridges between a positively (Arg, Lys) and a negatively (Asp, Glu) charged amino acid play an essential role in protein adaptation to extreme environments [97]. Here we observed a small but significant reduction in this kind of interaction in psychrophilic Clade Ib. This reduction has been suggested to play a role in weakening the structure of cold-adapted proteins and thus maintaining their flexibility [118, 119]. In contrast, in halophilic Clade II, we observed a substantial increase in salt bridges, which are known to contribute to the stability of proteins in high salinity conditions [120-122].

Stabilizing interactions that involve aromatic residues, namely, π–π, cation–π, and sulfur–π interactions, have been suggested to be important for environmental adaptation [97]. However, even though aromatic interactions in cold-adapted proteins are depleted in some families [123], the opposite trend is observed in other families [124]. In our present analysis, we did not find such a big difference between mesophilic and psychrophilic subclades in terms of interactions involving aromatic residues. There is, however, an increase in such interactions in the halophilic clades, but this is less intuitive if we think that large side chain residues are disfavored in high salinity conditions. However, the frequency of amino acids is not necessarily correlated with the frequency of their interactions.

With regard to hydrophobic interactions, we see a very small increase in Clade Ib with respect to Clade Ia, and this is consistent with the fact that the hydrophobic force is weaker at low temperature [125]. In the same vein, we observed an enrichment of hydrophobic interactions in thermophilic Clade IIb with respect to IIa, as hydrophobic interactions have also a reduced strength at higher temperature [125]. Finally, when comparing Clade IIa with Clade Ia to study the effect of halophilic environments, we observed an increase in hydrophobic contacts, which disagrees with previous observations [114] and the fact that high salt concentrations tend to enhance hydrophobic forces. This observed trend probably comes from the fact that not all hydrophobic residues are depleted in halophilic clades, as seen in the previous section: small hydrophobic residues, such as Val and Gly, tend to replace larger ones, such as Ile and Leu. Moreover, at high salt concentrations the overall shape of the protein structure is more compact, which further contributes to the increase in hydrophobic contacts [126].

To study how protein dynamics impact environmental adaptation, we used different indices that quantify the local protein flexibility and the conformational stability of the different clades. The first score we used was the SWOTein score *S* [55]. This per-residue index identifies stability strength and weakness regions in protein structure using the well-known statistical potential formalism [127]. Negative values represent residues that strongly contribute to the structural stability of the protein, whereas positive values indicate residues that are not optimized for stability, are more flexible, and are likely to play a functional role [55, 128].

There is a statistically significant difference between the average strength/weakness score in the different clades. In detail, Clade Ia is globally more stable than Clade Ib, with a decrease of the average *S* score of about 5% (<*S* > = −0.090 vs. −0.085 kcal/mol). This agrees with the fact that psychrophilic proteins are enriched in flexible regions, allowing enzymes to remain active at low temperatures. At high temperatures, we observed the opposite trend, as proteins tend to be more rigid [97, 99]. Indeed, we found that thermophilic Clade IIb has an average *S* that is bigger by about 10% than Clade IIa (<*S*> = −0.081 vs. −0.071 kcal/mol). Finally, when comparing Clades IIa and Ia, we observed an important drop in <*S*> of about 25% (<*S*> = −0.071 vs. −0.090 kcal/mol), which suggests that the proteins in the halophilic clade are weaker on average. This gives us a clear and interesting indication that weakness regions are not only important in psychrophiles, but also play an important role at high salt concentration, as has been suggested previously [105].

We also tested the predicted local distance difference test obtained as an output of the AlphaFold2 structure predictor [29] to quantify flexibility and stability, but did not find any difference between the clades, as described in Section S1.

### Family-dependent adaptation mechanisms

Adaptation mechanisms at protein level are usually family dependent [97, 99] and there is thus no universal mechanism that allows their adaptation to selective environments. To illustrate this, we provide an example of a family of homologous proteins in which we can clearly identify the complex interplay of adaptation strategies, which are difficult to observe at pangenome level where all effects are averaged. We focused here on pyruvate kinase (PYK), an important family of enzymes involved in carbohydrate metabolism. It catalyzes the last step of glycolysis, that is, the enzymatic reaction phosphoenolpyruvate + adenosine diphosphate → adenosine triphosphate + pyruvate [129].

**Figure 6:**
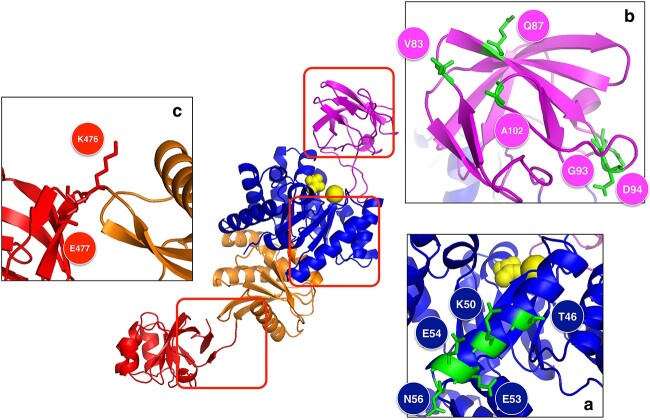
3D structure of PYK from *E. antarcticum* B7^T^. The complete structure is depicted in the middle. Panels (A–C) show the parts of the structure that contain residues that differ between mesophilic and halophilic homologs, and that play a key role in the adaptation of this protein to cold environments.

PYK has been found in prokaryotes and eukaryotes and is present in all *Exiguobacterium* isolates. The multiple sequence alignments (MSA) of PYKs from all isolates can be found in our repository github.com/environmental-genomes/Exiguobacterium. On a structural level, PYKs are composed of four domains, as shown in Fig. 6 for *E. antarcticum* B7^T^: a small ß-barrel Domain A and a larger aß-barrel Domain B, with the active site at the interface of these two domains; a three-layer αβα sandwich domain; and a mobile domain that binds to fructose 1,6-bisphosphate, the allosteric effector controlling the activity of PYK. Note that the biological unit of PYK is usually tetrameric, even though other oligomeric structures have been observed.

As an example of how protein structures can provide information about the environmental adaptation mechanisms used by the organism, we analyzed some of the key differences between subclades, paying particular attention to psychrophilic Subclade Ib. Although the protein sequence and structure are highly conserved between subclades, we focused on the variable regions and mapped them onto the 3D structure of *E. antarcticum* B7^T^ PYK. About 75% of the residue positions in the MSA are occupied by the same amino acid in all isolates, 17% of the positions share similar biophysical characteristics, and only 8% of the positions are occupied by dissimilar amino acids and thus allow PYK adaptation to different environments.

In the A domain, we noted two types of modifications (Fig. 6B). First, in and only in psychrophilic Clade Ib, three mutations (E83V, E87Q, and K102A) destroy three salt bridges. The decrease in the number of salt bridges is a known strategy to promote cold adaptation [97]. Second, a two-residue insertion in a loop (TE → GDAN at position 93) is observed in Clade Ia (mesophilic) and Ib (psychrophilic), but not in Clades IIa (halo-mesophilic) and Ib (halo-termophilic). The shortening of this loop can thus be interpreted as an adaptation mechanism to hotter environments [97], but also to saline environments.

In Domain B, there is a variable region corresponding to α-helix II (Residues 37–55) (Fig. 6A). It is more hydrophilic in Clade I (GRAVY index = −1.14) than in Clade II (GRAVY index = −1.04). Moreover, it is slightly less stable in Clade Ib than in Clade Ia, as predicted by SWOTein (in Clade Ia <*S*> = −1.05 kcal/mol; in Clade Ib <*S*> = −1.02 kcal/mol), whereas it is much stabler in Clade IIb (< *S* > = −1.4 kcal/mol) than in IIa (<*S* > = −0.95 kcal/mol). This helix, which is localized on the protein surface, is in direct contact with one of the loops involved in the catalytic site, and thus a more hydrophilic and less stable helix could increase the local flexibility to maintain the catalytic activity at low temperatures. In contrast, its strong stabilization at high temperature can greatly contribute to the heat resistance of this enzyme.

In the loop region connecting the C and D domains (Fig. 6C), we observe two charged residues (K476, E477), with the former forming a salt bridge with a residue of Domain C and the latter, with a residue of Domain D. In halophilic Clade II, K476 is replaced by a histidine, and E477 by an alanine, which breaks the salt bridge interaction. The salt bridge is nevertheless recovered in halo-thermophilic Clade IIb, where N480 is replaced by an aspartic acid, known to be preferred at high temperatures [97].

The PYK family is an example of highly conserved proteins that have adapted to completely different environments with sequences that differ by just a few residues. Our structural analysis helps to better understand these adaptation strategies.

## Discussion

Here we have shown how integration of a wide range of information at different levels, from nucleotide to protein 3D structure, can contribute to the definition of sharply delineated ecological units. Indeed, we combined phylogenomic information, genomic features, gene and pathway enrichment data, regulatory element analysis, protein amino acid composition, protein structural data, and experimental growth curve tests, and this allowed us to resolve the conflicts between phylogeny and isolation source and to shed light on the genetic basis of the defined ecotype. The main findings of our study are summarized and discussed below.

### Ordering *Exiguobacterium* into ecologic units

Determining the genetic basis of an ecotype is one of the most pervasive objectives in microbiology [79, 130]. However, habitat-specific functions are often elusive in taxon or phylogenetic clades [69]. Furthermore, bacteria from different habitats tend to form mixed lineages, or, in contrast, isolates from the same habitat separate on the phylogenetic tree, making it hard to delineate monophyletic ecological units belonging to specific environmental conditions [4, 68]. Indeed, the core group of dominant phylotypes that prevail across global soils are without clearly identifiable habitat preferences [131].

To overcome the difficulties in mapping the vast microbial diversity into ecologically meaningful units, we have introduced a new approach that, alongside phylogenomic and functional analyses, also considers genome-wide regulatory features and pangenome protein structure data. Even if our work was inspired by the reverse ecology approach, our main focus was on mapping genomes (including individual genomes and MAGs) from similar but geographically distant habitats into ecological units, rather than requiring them to coexist in the same environments or hosts [6].

We have succeeded in delineating all 78 *Exiguobacterium* genomes into ecological units at two phylogenomic resolutions. The metadata-based classification divided *Exiguobacterium* into two main ecotypes: nonsaline Clade I and saline Clade II; and four sub-ecotypes: nonsaline mesophilic Clade Ia, nonsaline psychrophilic Clade Ib, saline mesophilic Clade IIa, and saline thermophilic Clade IIb. It was previously suggested that the formation of Clades I and II is mainly related to the growth temperature profile [14, 17, 132]. However, our results indicate that the division of Clades I and II mainly corresponds to the salinity of the habitat, whereas temperature resistance is related to the division of clades into subclades.

With this grouping scheme for the *Exiguobacterium*, unification of phylogenetic, genomic, phenotypic, and isolation information was maximally achieved and we resolved the contrast between the isolation source and the phylogeny [14]. For example, isolates from nonpolar environments, such as the strain *Exiguobacterium oxidotolerans* JCM 12280^T^, which were isolated from fish-processing plants, were classified as a cold-adapted ecotype, given that they share conserved genomic features with polar isolates and were clustered together with them. On the same basis, we assigned *Exiguobacterium* sp. GIC31 to a halophilic ecotype rather than a psychrophilic one and *Exiguobacterium indicum* HHS 31 to a mesophilic ecotype, even though they were both isolated from glaciers. Indeed, strain *E. indicum* HHS 31 grows weakly at 2.5°C and is unable to grow at subzero temperatures [14, 133]. In fact, the assembly of glacier (and permafrost) microbial communities is determined by both long-time adaptational evolution and contemporary dispersal [9]. For the dispersed isolates (e.g. *Exiguobacterium* sp. GIC31), there may not be enough time for them to adapt to the new selective environmental conditions and just survive via physiological plasticity [24], or the isolates may not be able to colonize at all; thus, they do not share the same psychrophilic ecotype with isolates in Clade Ib [8, 9]. This situation also applied to the Antarctic lake strain *Exiguobacterium* sp. U13-1; another strain, *Arthrobacter* sp. U41, from the same lake, was also not in the cold adapted clade as shown in a previous study [78]. It is important to remember that low-temperature conditions are ideal for preserving DNA and cells, so not all glacier-derived isolates necessarily belong to the cold adapted ecology type [134, 135]. The grouping scheme is also applied to MAGs whose physiological properties are difficult to detect. Most of the New York subway MAGs (7/8) belong to the nonsaline Clade I, but one of them (*Exiguobacterium* sp. UBA4960) was assigned to the saline Clade II. Note that even though clades are optimized for given environmental conditions, they can usually withstand large fluctuations thanks to their high phenotypic plasticity [27, 136], the extent of which varies between clades (see Table S1).

### New mechanisms of microbial environmental adaptation

The analysis of genome-wide regulatory features sheds light on new mechanisms used by bacteria to selectively adapt to different environments. Numerous studies have been performed to identify genes that are critical for habitat adaptation of extremophiles: e.g. osmotic stress response genes in saline adaptation [137], pigment synthesis genes in UV resistance [138], and DNA repair genes in radio-resistance [75]. However, little attention has been paid to the regulatory elements of the extremophiles, leaving a critical knowledge gap in fully decoding environmental adaptation [82]. Most genes do not regulate themselves, but their function depends on regulatory elements, such as sRNA, promoters, and enhancers. Here we have shown that regulatory elements play an important role in environmental adaptation and should be more properly considered in environmental microbial genomic and metagenomic studies. For example, in the psychrophilic clade, we observed a significant decrease in GC content of tmRNA, an improved transcription efficiency by promoter modification, an increased density of operon per CDS, and an enrichment in sRNAs.

Experimental methods to detect and verify the role of regulatory elements in bacterial environmental adaptation are labor intensive and expensive, and their outputs require exhaustive manual correction, often inapplicable at the genome scale [48, 130]. We show that the analysis of regulatory elements and their association to genotype-ecotype relationship is an ingenious way of using sequencing data to discover complex patterns of bacterial adaptation and to deeper understand the genetic basis of ecotypes [130].

Our pangenome protein structure analysis provides new insightful information on adaptation mechanisms for extreme environments. In more detail, this extensive investigation points out the prominent role of protein surface residues, which contribute much more than core residues to the environmental adaptation. Protein cores are relatively well conserved between subclades, whereas amino acid changes occur preferentially at the protein surface. This follows from the fact that surface residues are less constrained and evolve faster than core residues [139], and are thus more easily substituted for environmental adaptation. Our structural analysis also allowed the identification of the type of amino acid interactions that contribute more than others to adaptation, such as salt bridges, and illustrated how sequence differences of just few residues can lead to the adaptation to completely different environments, taking the PYK family as an example.

Note that the structural analysis was essential to rationalize the observed trends and to better understand the adaptation strategies. We would like to underline that this analysis was made possible by the recent development of accurate structure prediction methods. The present study paves the way for the use of large-scale protein structure information in microbial ecology and evolution.

### Improving the extrapolation of genomic knowledge of environmental relevance

Our results show that it is possible to assign ecotype to every single isolate or MAG of a microbial taxa, at least at a coarse grain level. Then, based on the reverse ecology principle, genetic adaptations of microorganisms to their native environment, as well as their response to changing environmental conditions, can be more precisely investigated. For example, we identified sulfur cycling genes that are specifically enriched in the psychrophilic clade. As temperature increases, the activity of psychrophiles increases until some process in the cell becomes thermally compromised [140]. We can thus predict that increasing temperature would impact sulfur cycling mediated by psychrophilic *Exiguobacterium* spp. in a nonlinear way. This supports the hypothesis that the effect of climate change on environmental microbes and their response will not show a linear increase or decrease over ecological time [141].

Moreover, by analyzing the ratio of subclades in an environment, we can predict the functional changes of the *Exiguobacterium* microbial community. For example, we hypothesize that an increase in the ratio of Clade I–II indicates an enhancement of the degradation ability of organic matter, and that an increase in the ratio of Clade Ia–Ib suggests microbial responses to higher temperatures and a depletion in sulfur metabolism. A similar speculation on how change in environmental conditions led to functional changes in microbial communities has been verified in Shen *et al*. [78] by analyzing large amounts of metagenomes and studying the temperature response of mesophilic *Arthrobacter* in polar and alpine samples.

In summary, we showed that elucidating the genetic basis of environmental microbial units can facilitate the prediction of microbial responses to climate change (e.g. melting glaciers and permafrost, soil salinization because of drought), and pointed out the important role that genomic data can play in climate models [7, 8, 141].

## Supplementary Material

Table_S1_wrad020

Table_S2_wrad020

Table_S3_wrad020

R2_Supplementary_Material_Exiguo_17_7_3_wrad020

## Data Availability

All data and code from this paper are available in our repository github.com/environmental-genomes/Exiguobacterium.
