# Supplementary material for: Genomic basis of environmental adaptation in the widespread poly-extremophilic Exiguobacterium group
Source: ISME J. 2024 Jan 10;18(1):wrad020. doi: 10.1093/ismejo/wrad020 (PMC10837837; doi:10.1093/ismejo/wrad020)
Supplement: R2_Supplementary_Material_Exiguo_17_7_3_wrad020 [file r2_supplementary_material_exiguo_17_7_3_wrad020.pdf]

Supplementary Material

The genomic basis of environmental adaptation in the widespread poly-extremophilic *Exiguobacterium* group

Liang Shen[1,2], Yongqin Liu[3], Liangzhong Chen[1], Tingting Lei[1], Ping Ren[1], Mukan Ji[3], Weizhi Song[4], Hao Lin[5], Wei Su[5], Sheng Wang[6], Marianne Rooman[7,8], Fabrizio Pucci[7,8*]

[1]College of Life Sciences, Anhui Normal University, Wuhu 241000, China

[2]Anhui Provincial Key Laboratory of Molecular Enzymology and Mechanism of Major Diseases, and Anhui Provincial Engineering Research Centre for Molecular Detection and Diagnostics, Anhui Normal University, Wuhu 241000, China

[3]Center for the Pan-third Pole Environment, Lanzhou University, Lanzhou 730000, China

[4]Centre for Marine Bio-Innovation, University of New South Wales, Sydney,

NSW 2052, Australia

[5]School of Life Science and Technology, University of Electronic Science and Technology of China, Chengdu 611731, China

[6]Shanghai Zelixir Biotech Company Ltd., Shanghai 200030, China

[7]Computational Biology and Bioinformatics, Université Libre de Bruxelles, Brussels 1050, Belgium

[8]Interuniversity Institute of Bioinformatics in Brussels, Brussels 1050, Belgium

*Correspondence: Fabrizio.Pucci@ulb.be

- **Fig. S1.** *Exiguobacterium* phylogeny based on 16S rRNA gene.
- **Fig. S2.** Growth test of *Exiguobacterium* at different salinity.
- **Fig. S3**. GC content of *Exiguobacterium*
- **Fig. S4. Horizontal gene transfer events detected in** *Exiguobacterium*
- **Fig. S5.** Genome compositional features of the four *Exiguobacterium* subclades.
- **Fig. S6.** Heat map of carbohydrate-active enzyme gene classes and cold shock genes along with Exiguobacterium phylogenetic tree.
- **Fig. S7. Analysis of carbohydrate substrates utilization profile of the *Exiguobacterium***
- **Section S1.** Local flexibility and conformational stability.
- **Fig. S8.** Comparison of the amino acid composition of surface regions in the four subclades.
- **Fig. S9.** Comparison of the amino acid composition of core regions in the four subclades.

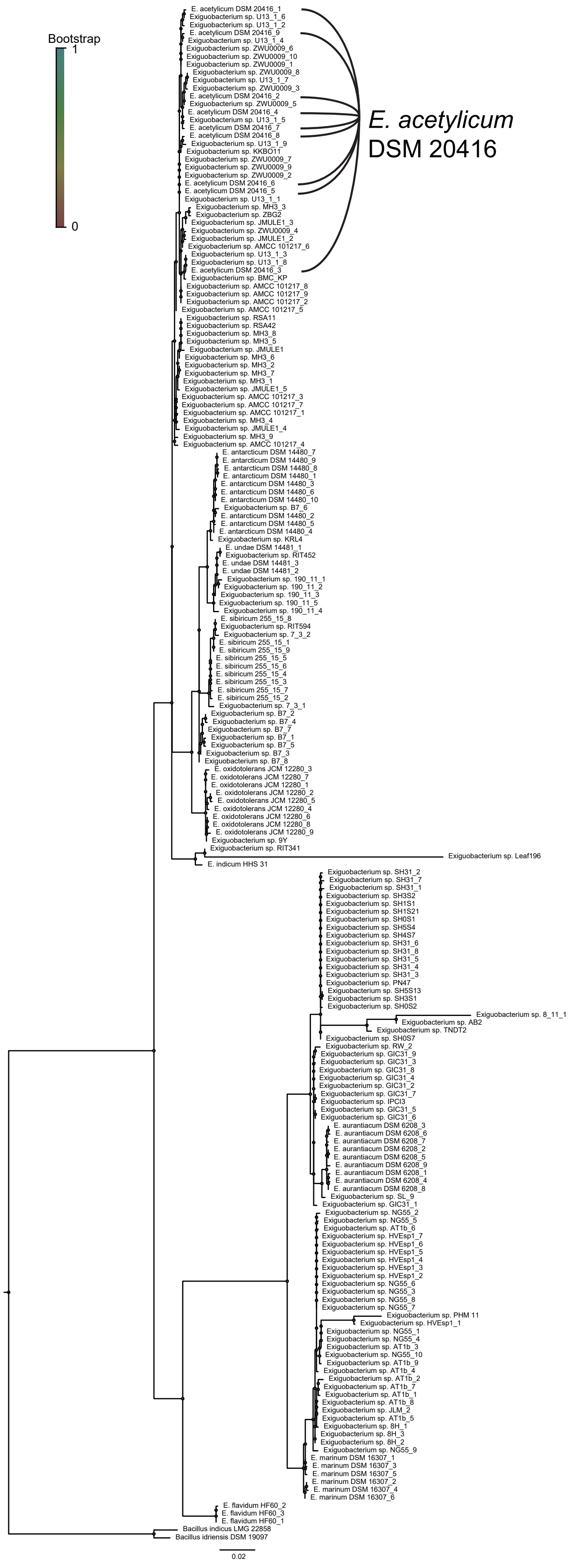

Fig. S1. *Exiguobacterium* phylogeny based on the 16S rRNA gene. Multiple divergent rRNA resulting in mobile positioning of a strain (i.e. *E. acetylicum* DSM 20416) in the phylogenetic tree. Bar 0.02 accumulated changes per nucleotide.

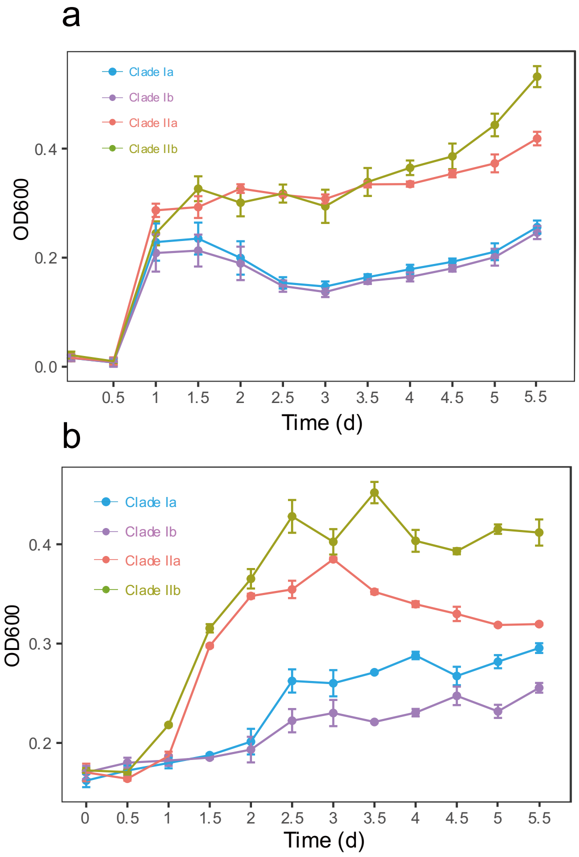

Fig. S2. Growth test of *Exiguobacterium* at different salinity. Growth curves for representative isolates of clade Ia (blue symbols and line), clade Ib (purple symbols and line), clade IIa (orange symbols and line) and clade IIb (olive green symbols and line) at (a) 3% and (b) 5% NaCl (m/v) at 25 °C.

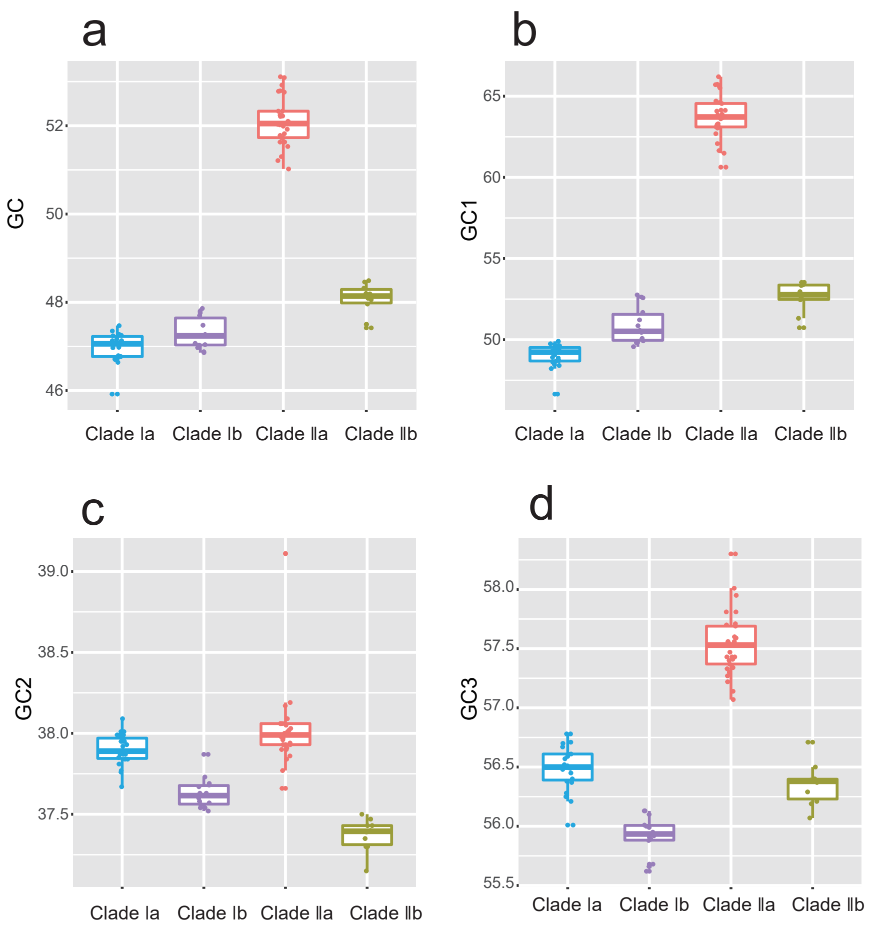

Fig. S3. GC content of *Exiguobacterium*. GC content: (a) genome-wide, (b) at first codon position in the exome (GC1), (c) at second codon position in the exome (GC2), (d) at third codon position in the exome (GC3).

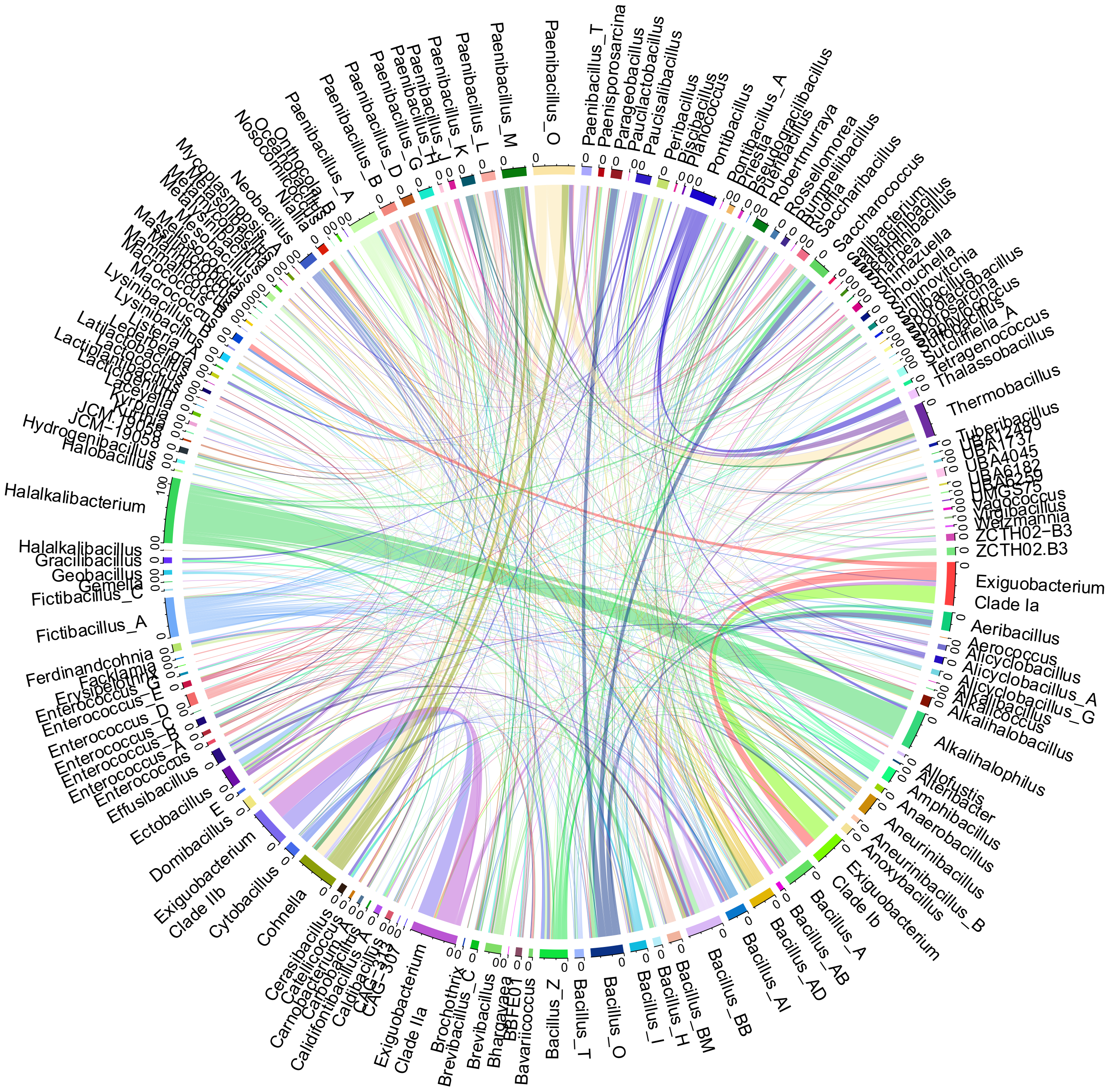

Fig. S4. Horizontal gene transfer events detected in *Exiguobacterium*. The 78 *Exiguobacterium* genomes, together with 337 representative genomes sampled from each species of the order Bacilli in the GTDB database, were provided as input to MetaCHIP.

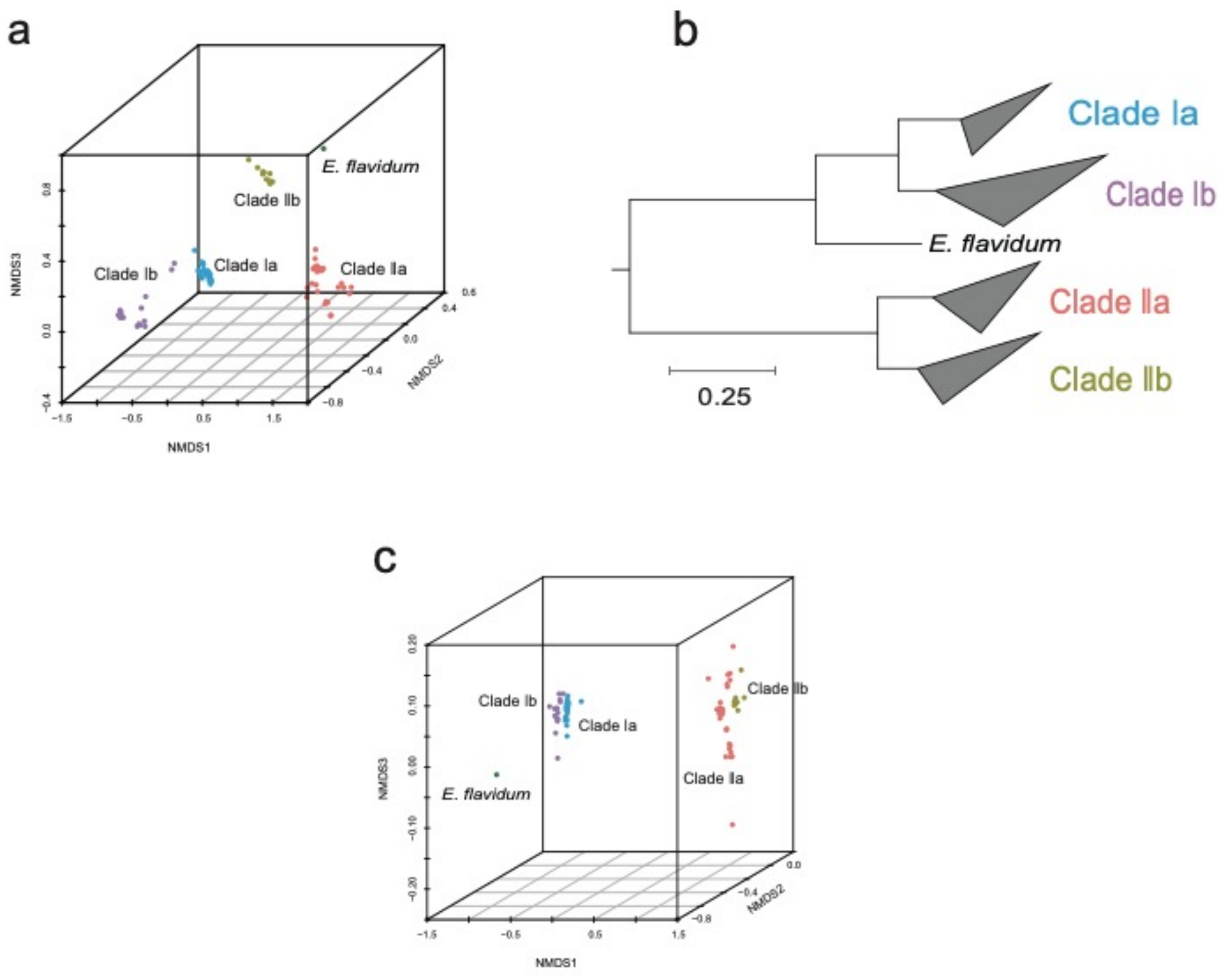

Fig. S5. Genome compositional features of the four *Exiguobacterium* subclades. (a) Non-metric multidimensional scaling (NMDS) plots of genome-wide codon usage. (b) Recovering of the four sub-clades at the same amount of evolutionary distance; the threshold for groups (grey wedges) was an average branch length of < 0.65 substitutions per site. (c) NMDS plots of about 4500 functional genes identified by PROKKA.

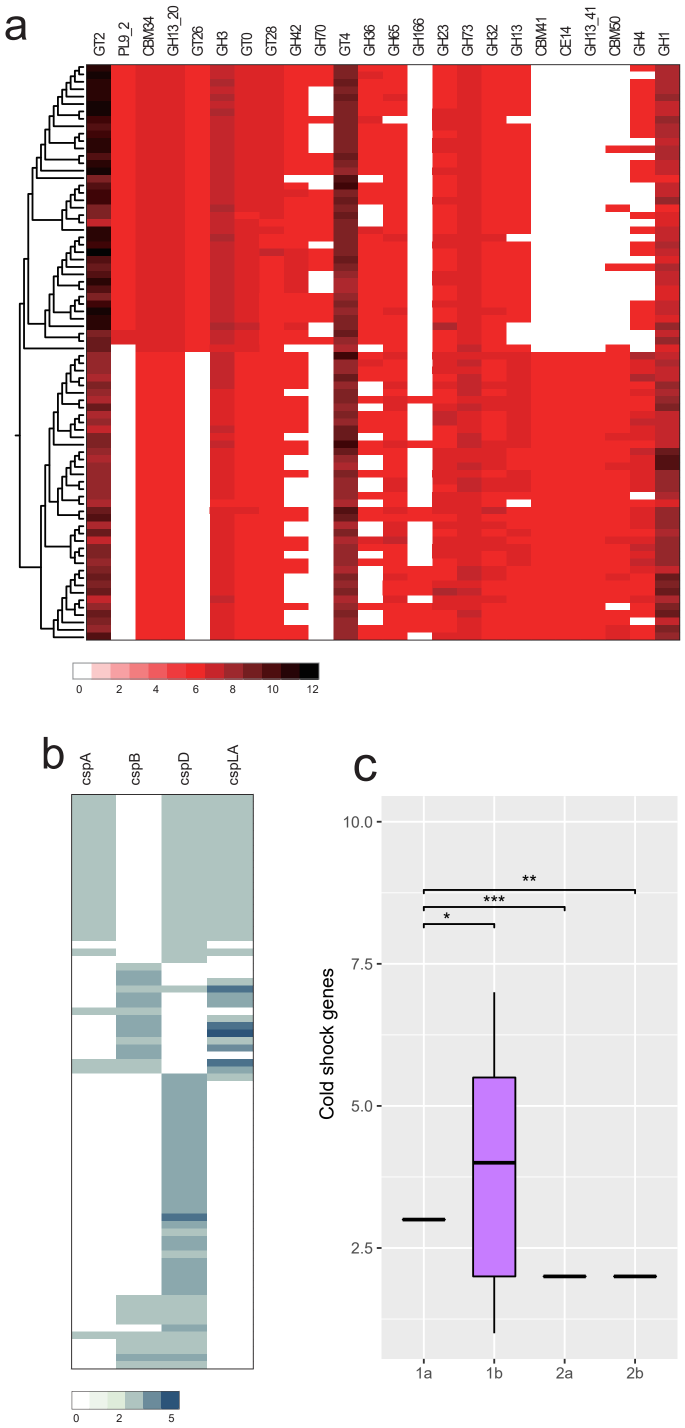

Fig. S6. Heat map of (a) carbohydrate-active enzyme gene classes and (b) cold shock genes along with *Exiguobacterium* phylogenetic tree. (c) Average number of cold shock genes for each subclades.

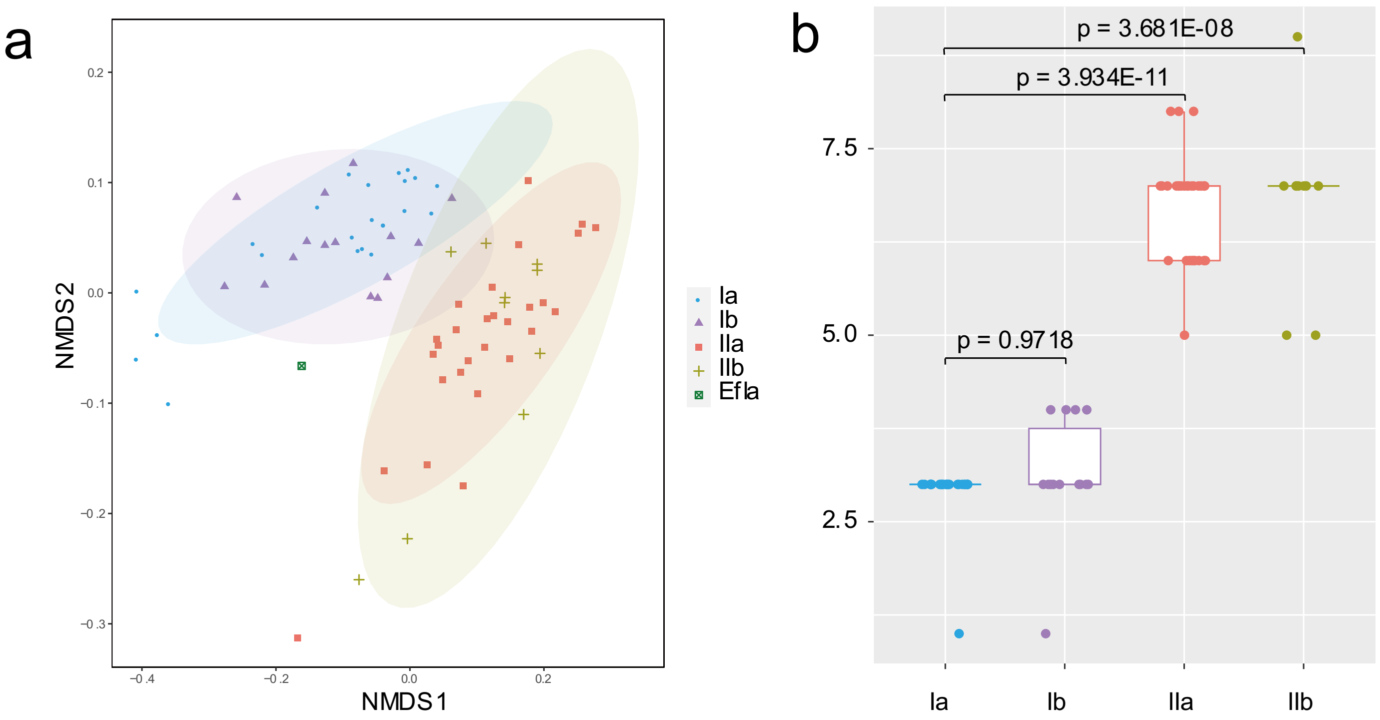

Fig. S7. Analysis carbohydrate substrates utilization profile of the *Exiguobacterium*. (a) NMDS analysis of carbohydrate substrates utilization profile of *Exiguobacterium* clades. (b) Boxplot showing the distribution of CAZymes linked to chitin utilisation among the four *Exiguobacterium* clades.

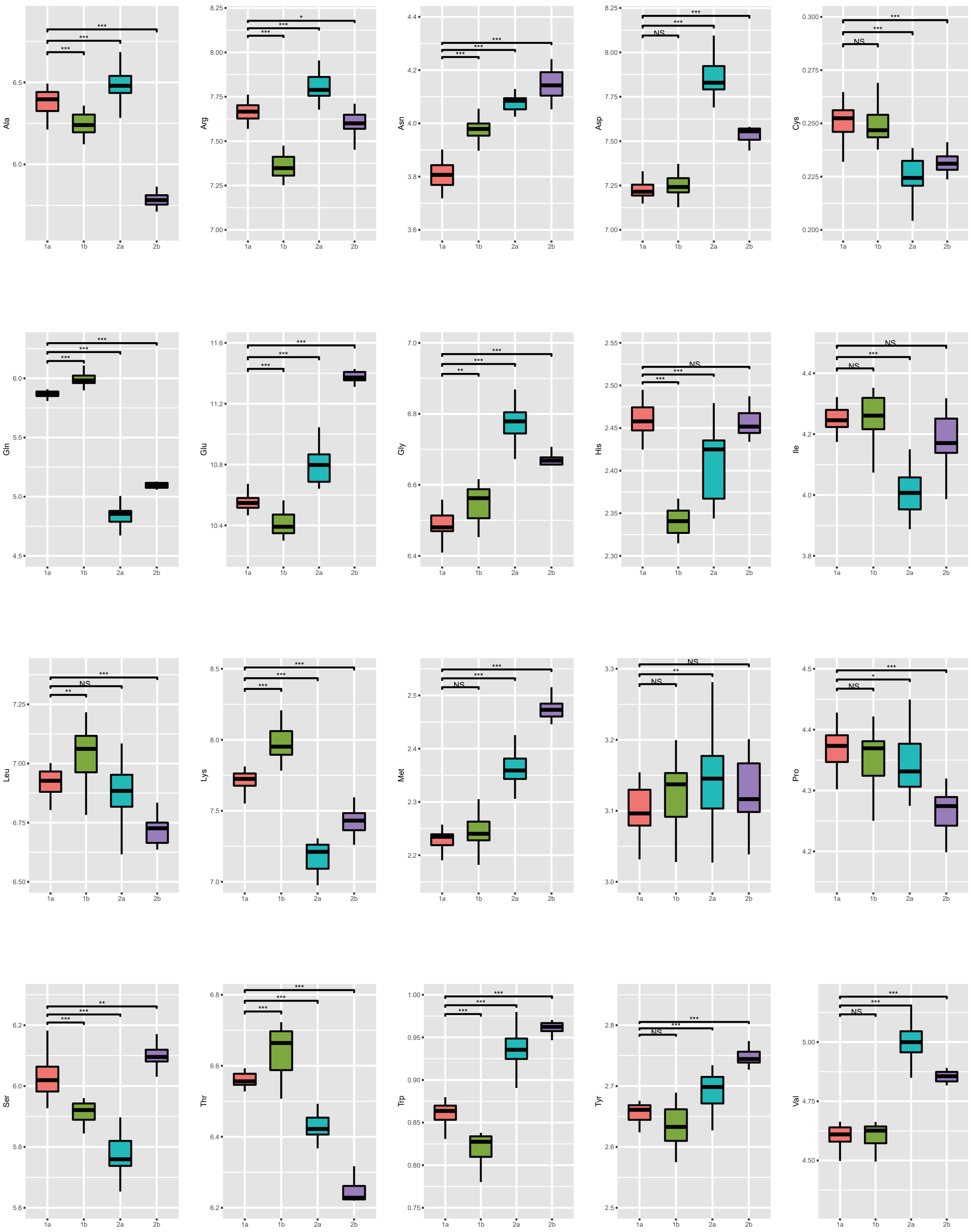

Fig. S8. Comparison of the amino acid composition of surface regions in the four subclades. *$p < 0.05$; **$p < 0.01$; ***$p < 0.005$, NS, not significant ($p > 0.05$).

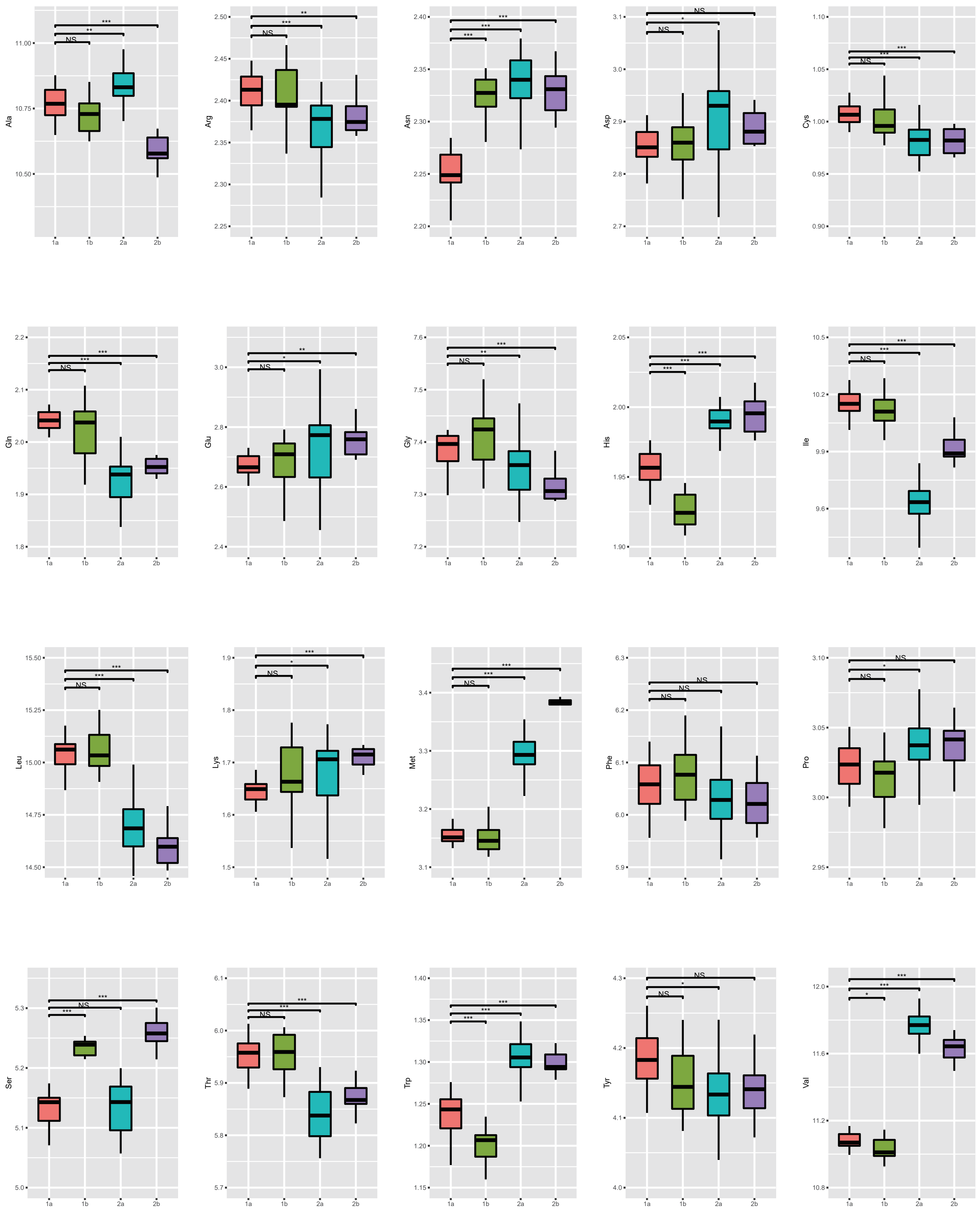

Fig. S9. Comparison of the amino acid composition of core regions in the four subclades. *$p < 0.05$; **$p < 0.01$; ***$p < 0.005$, NS, not significant ($p > 0.05$).

Section S1. Local flexibility and conformational stability

To study how protein dynamics impact environmental adaptation, we used different indices that quantify the local protein flexibility and the conformational stability. One of these indices is the predicted local distance difference test (pLDDT), obtained as an output of the AlphaFold2 structure prediction tool [1]. Indeed, it has been shown that this score is well correlated with the residue root mean square fluctuations, as calculated from molecular dynamics simulations, and can thus, in principle, be used as a proxy for identifying flexible (low pLDDT) and rigid (high pLDDT) regions in protein structures [1,2]. However, it should be noted that this score is also low when no homolog of the target protein is found, and thus lower values do not always correspond to flexible regions, but to incorrectly predicted ones.

We did not observe any substantial difference in the pLDDT distribution between the different clades. All clades have a mean pLDDT value of about 90, which means that the vast majority of the *Exiguobacterium* pangenome is predicted by AlphaFold2 to be well structured, with high confidence. In contrast, we did find differences between the local stability properties in the different clades using the SWOTein score [3], as described in the main text.

[1] Jumper, J., Evans, R., Pritzel, A., Green, T., Figurnov, M., Ronneberger, O. et al. (2021) Highly accurate protein structure prediction with AlphaFold. *Nature* **596**: 583-589.
[2] Guo, H.B., Perminov, A., Bekele, S., Kedziora, G., Farajollahi, S., Varaljay, V. et al. (2022) AlphaFold2 models indicate that protein sequence determines both structure and dynamics. *Sci Rep* **12**: 10696.
[3] Hou, Q., Pucci, F., Ancien, F., Kwasigroch, J.M., Bourgeas, R., and Rooman, M. (2021) SWOTein: a structure-based approach to predict stability strengths and weaknesses of proteins. *Bioinformatics* **37**: 1963-1971.
